# Meiotic chromatin-associated HSF5 is indispensable for pachynema progression and male fertility

**DOI:** 10.1093/nar/gkae701

**Published:** 2024-08-20

**Authors:** Chunhai Luo, Haoran Xu, Ziqi Yu, Dalin Liu, Danyang Zhong, Shumin Zhou, Beibei Zhang, Junfeng Zhan, Fei Sun

**Affiliations:** Department of Urology & Andrology, Sir Run Run Shaw Hospital, Zhejiang University School of Medicine, Hangzhou, Zhejiang 310016, China; Department of Urology & Andrology, Sir Run Run Shaw Hospital, Zhejiang University School of Medicine, Hangzhou, Zhejiang 310016, China; Department of Urology & Andrology, Sir Run Run Shaw Hospital, Zhejiang University School of Medicine, Hangzhou, Zhejiang 310016, China; Department of Urology & Andrology, Sir Run Run Shaw Hospital, Zhejiang University School of Medicine, Hangzhou, Zhejiang 310016, China; Department of General Surgery, Sir Run Run Shaw Hospital, Zhejiang University School of Medicine, Hangzhou, Zhejiang 310016, China; Department of Urology & Andrology, Sir Run Run Shaw Hospital, Zhejiang University School of Medicine, Hangzhou, Zhejiang 310016, China; Department of Urology & Andrology, Sir Run Run Shaw Hospital, Zhejiang University School of Medicine, Hangzhou, Zhejiang 310016, China; Department of Urology & Andrology, Sir Run Run Shaw Hospital, Zhejiang University School of Medicine, Hangzhou, Zhejiang 310016, China; Department of Urology & Andrology, Sir Run Run Shaw Hospital, Zhejiang University School of Medicine, Hangzhou, Zhejiang 310016, China

## Abstract

Pachynema progression contributes to the completion of prophase I. Nevertheless, the regulation of this significant meiotic process remains poorly understood. In this study, we identified a novel testis-specific protein HSF5, which regulates pachynema progression during male meiosis in a manner dependent on chromatin-binding. Deficiency of HSF5 results in meiotic arrest and male infertility, characterized as unconventional pachynema arrested at the mid-to-late stage, with extensive spermatocyte apoptosis. Our scRNA-seq data confirmed consistent expressional alterations of certain driver genes (*Sycp1*, *Msh4*, *Meiob*, etc.) crucial for pachynema progression in *Hsf5*^−/−^ individuals. HSF5 was revealed to primarily bind to promoter regions of such key divers by CUT&Tag analysis. Also, our results demonstrated that HSF5 biologically interacted with SMARCA5, SMARCA4 and SMARCE1, and it could function as a transcription factor for pachynema progression during meiosis. Therefore, our study underscores the importance of the chromatin-associated HSF5 for the differentiation of spermatocytes, improving the protein regulatory network of the pachynema progression.

## Graphical Abstract

**Graphical Abstract:**
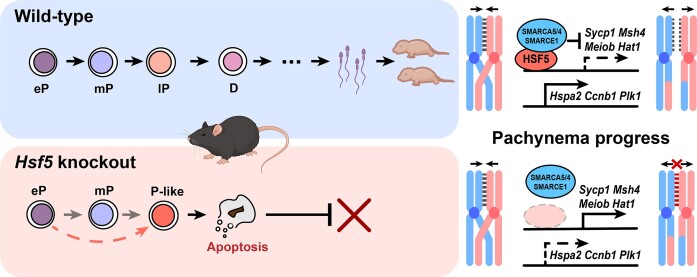

## Introduction

Meiosis, an essential event in gametogenesis, entails a single round of DNA replication followed by two rounds of cell division (meiosis I and II), culminating in the production of four haploid gametes. After completing chromosome replication during the premeiotic S phase, primary germ cells enter Meiotic Prophase I, a prolonged G2 phase that guarantees the completion of numerous meiosis-specific chromosome events (1). Prophase I is comprised of five distinct substages, namely leptonema, zygonema, pachynema, diplonema, and diakinesis. These substages are characterized by specific features, including programmed DNA double-strand breaks (DSBs) generation as well as the formation and disassembly of the synaptonemal complex (SC) (2,3). The SC serves as a tripartite protein scaffold crucial for facilitating pairing and DNA double-strand break repair of homologous chromosomes (4,5). During leptonema, homologs align between axial elements composed of SYCP2, SYCP3 and cohesin. Subsequently, in zygonema, central elements facilitate the physical connection between pairs of homologs. The process progresses to pachynema, where synapsis of homologs is completed. Gradual disassembly of the SC occurs during diplonema. Finally, in diakinesis, homologs recondense as the transition to metaphase ensues (6).

During prophase I, the pachytene stage stands out as the longest among the five stages, underscoring its unique and enigmatic role in meiosis (7). In recent decades, significant attention has been devoted to investigating the molecular mechanisms underlying the pachynema progression. While numerous mutant mouse models and transcriptomic studies have been developed to explore key events in the pachytene stage, such as *Hspa2*, *Maps*, *Zfp541* (8–13), the protein regulatory network or key factors governing pachynema progression remain incompletely understood (14,15).

Heat shock factors (HSFs) play a critical role in regulating the quality of cellular proteins by binding specifically to the heat shock element (HSE) in the genome, a process known as the unfolded protein response (UPR) or heat shock response (HSR) (16–18). They achieve this by controlling the expression of numerous genes, including those encoding inducible protein chaperones or heat shock proteins (HSPs) (18,19). The HSFs family comprises several paralogs: HSF1-5, HSFY and HSFX, some of which have been reported to participate in spermatogenesis (17). Either heat shock transcription factor family 1 (HSF1) or heat shock transcription factor family 2 (HSF2) deficient mice exhibit mildly spermatogenetic defects but are still fertile (20). Double knockout of HSF1 and HSF2 leads to mice infertile, of which spermatogenesis arrests at pachytene stage (21).

The fifth member of the heat shock factor family (HSF5), a hitherto not fully characterized member of the heat shock factor family, is a non-canonical HSF that possesses a winged-helix-turn-helix (WHTH)-like DNA-binding domain and is specifically expressed in the testis (17,18). Previous studies have reported that HSF5 is required for zebrafish spermatogenesis, as evidenced by a reduction in sperm count and abnormal tail architecture upon HSF5 disruption (22). During the course of our study, two different works have mentioned the importance of HSF5 for spermatogenesis, but their results of phenotypic analysis and mechanistic explanations vary a lot (23,24). Here, through the innovative chromatin-associated protein screening method, we identified a novel candidate HSF5, loss of which leads to severe meiotic arrest, abnormal pachynema with accumulated apoptotic spermatocytes, and eventually male infertility. Combined with multi-omics integrative analysis, such as the integration of 10x Genomics single-cell RNA-seq (scRNA-seq), and Cleavage Under Target & Tagmentation (CUT&Tag) techniques, HSF5 was characterized as a transcriptional factor of driver genes essential for pachynema progression, achieved by its interaction with SMARCA5, SMARCA4, and SMARCE1 and binding to promoter regions of genes whose transcriptional programs occur in orderly repressive or inductive manners. In summary, our findings revealed a master molecular mechanism of HSF5 in the controlling network of pachynema progression.

## Materials and methods

### Isolation of spermatogenic cells (STA-PUT method)

Three types of mouse male germ cells, namely pachytene spermatocytes (SPC), round spermatids (RS) and elongating spermatids (EST), were isolated from certain testes using a BSA gradient method as previously described (25). Upon euthanasia, eight mouse testes were harvested, swiftly washed twice in cold PBS, and subsequently digested in 10 ml Krebs–Ringer solution (K4002, Sigma) supplemented with collagenase (0.5 mg/ml, 10103578001, Sigma) at 33°C for 15 min with gentle agitation. The dispersed seminiferous tubules were then washed twice with Krebs–Ringer solution. Following this, the tubules underwent digestion in 20 ml Krebs–Ringer solution containing trypsin (0.5 mg/ml, T8003, Sigma) and DNase I (1 mg/ml, DN25, Sigma) at 33°C for 10 min with gentle agitation, followed by filtration through a 40 μm Nylon Cell Strainer (352340, BD Falcon) and resuspension in 25 ml Krebs–Ringer solution containing 0.5% bovine serum albumin (BSA) (A600332, Sangon Biotech). Subsequently, the single-cell suspension was loaded into the separation apparatus (ProScience, Canada), and germ cell populations were separated by sedimentation at unit gravity for 3 hours through a gradient of 2–4% BSA solution. Following sedimentation, cell fractions were harvested and identified based on their morphological characteristics. The purity of SPC, RS, and EST populations was approximately 80%, 90% and 85%, respectively, determined by analysis using an Olympus BX43 microscope and immunofluorescence analysis.

### Purification of meiosis chromatin-associated protein

This method was adapted from our prior research (26). Initially, spermatocytes purified by STA-PUT method or whole testes were homogenized in Buffer A (250 mM sucrose, 10 mM Tris–HCl, pH 8.0, 10 mM MgCl_2_, 1 mM EGTA, 1x protease inhibitor cocktail), with a portion reserved as whole cell lysate for subsequent Western blotting (WB) analysis. Subsequently, nuclei were pelleted by centrifugation at 300g at 4°C for 5 min, and the supernatant underwent another high-speed centrifugation at 14 000g at 4°C for 10 min to obtain purified cytoplasmic proteins. The resulting pellet was homogenized in Buffer B (250 mM sucrose, 10 mM Tris–HCl, pH 8.0, 10 mM MgCl_2_, 1 mM EGTA, 0.1% Triton X-100, 1% NP-40, and 1× protease inhibitor cocktail) and then centrifuged at 300g at 4°C for 3 min to remove nuclear membrane debris. Following this, the chromatin-containing supernatant was carefully layered over a 1.7M sucrose cushion and centrifuged at 50 000g for 1 h in a Beckman SW32Ti rotor. The resulting pellets were resuspended in Buffer B, then centrifuged at 1300g to precipitate the chromatin, which was subsequently snap-frozen and stored at –80°C for analysis using high-resolution mass spectrometry (HRMS) or WB.

### Antibodies

To generate the antibody targeting mouse HSF5, mouse *Hsf5* cDNA spanning nucleotides 1282–1875 was inserted in-frame into the Pet42b vector, resulting in the fusion protein (HSF5-8x His) comprising C-terminal amino acids 428–624 of mouse *Hsf5* and eight tandem histidine residues. The fusion protein was expressed in Escherichia coli (strain BL21), subsequently subjected to affinity purification using Ni-NTA (nitrilotriacetic acid) resin, and utilized for immunization of mice, rabbits, and Norway rats with FCA (KX0210046Q, Biodragon). Antibodies were subsequently purified using an antigen affinity column. The other antibodies involved are listed in Supplementary Table S1.

### Mice and genotyping

All animal procedures were approved by the Zhejiang University Institutional Animal Care and Research Committee, and mouse care was performed in accordance with the relevant guidelines and regulations of Zhejiang University. The *Hsf5* heterozygous (*Hsf5*^+/−^) mouse strain on a C57BL/6 background, created utilizing CRISPR/Cas9 technology, was procured from the Shanghai Model Organisms Center, Inc. The KO methodology is detailed in Figure 2A. Founder mice genomic DNA was scrutinized via PCR and sequencing to confirm the *Hsf5* gene mutation. To mitigate potential off-targeting effects, *Hsf5*^+/−^ mice were backcrossed with wild-type (WT) mice for three successive generations. All mutant mouse strains maintained a C57BL/6 background. Guide RNAs (gRNAs) are provided in Supplementary Table S1. Standard specific pathogen-free (SPF) conditions were maintained for housing all mice, comprising a temperature range of 20–22°C, a 12 hours light/dark cycle, humidity maintained at 50–70%, and an ample supply of food and water. Mutant mice genotyping was performed by PCR amplification (HRF0030, Fujian Herui Biological Technology Co., Ltd) of genomic DNA extracted from mouse tail tips (12). Genotyping PCR primers are listed in Supplementary Table S1.

### Fertility test

For the male fertility assessment, adult *Hsf5*^−/−^ males and their *Hsf5*^+/+^ male littermates were mated with two adult WT females each. For the female fertility assessment, two adult *Hsf5*^−/−^ females and their *Hsf5*^+/+^ female littermates were each mated with one adult WT males. Breeding was sustained continuously for a minimum of 3 months. Throughout the fertility evaluation period, vaginal plugs were inspected daily. The occurrence of vaginal plugs, instances of pregnancy, and the count of offspring were documented to determine the pups per plug and pups per litter ratios.

### Histological, immunofluorescence and TUNEL staining in testicular sections

Testes or epididymides from *Hsf5*^+/+^ and *Hsf5*^−/−^ mice were dissected, fixed with either Bouin's solution or 4% paraformaldehyde (PFA), subsequently embedded in paraffin, and sectioned at a thickness of 5 μm for subsequent staining. Sections fixed with Bouin's solution were stained using standard protocols for hematoxylin and eosin (H&E) as well as Periodic Acid-Schiff (PAS) reagent (G1285, Solarbio). H&E and PAS images were captured by using a light microscope (Olympus BX43). Immunofluorescence studies were conducted on sections fixed with 4% PFA, which were subjected to dewaxing, rehydration, and antigen retrieval by incubation with sodium citrate buffer (pH 6.0) at 95°C for 15 min. Nonspecific antigens were blocked using 5% donkey serum (GTX30972, GeneTex) for 1 h at room temperature. Primary antibodies were diluted with 5% donkey serum and subsequently incubated with sections at 4°C overnight. Following three washes with phosphate-buffered saline (PBS), sections were incubated with diluted secondary antibodies conjugated with a fluorescent tag or Cy3-conjugated peanut agglutinin (PNA) for 2 hours at room temperature. sections were incubated with secondary antibodies diluted and conjugated with a fluorescent tag, or with Cy3 PNA, for 2 hours at room temperature. The nucleus was stained with 4′,6-diamidino-2-phenylindole (DAPI) diluted in PBS, and photomicrographs were captured using confocal fluorescence microscopes (LSM980, Zeiss). TUNEL staining was conducted using the Dead End Fluorometric TUNEL System (KGA1408-20, Keygen BioTECH) according to the manufacturer's protocol. The seminiferous epithelium cycle comprises 12 stages (I to XII) delineated by the development of spermatocytes and spermatids (27).

### Spermatocyte spreading and immunofluorescence staining

*Hsf5* ^+/+^ and *Hsf5*^−/−^ testes were dissected, and the seminiferous tubules were washed in PBS. The tubules were then incubated in a hypotonic extraction buffer (50 mM sucrose, 17 mM trisodium citrate dehydrate, 5 mM EDTA, 0.5 mM DTT and 1× protease inhibitor cocktail, pH 8.2) for 45 min on ice. Subsequently, the tubules were minced in 100 mM sucrose (pH 8.2) on a clean glass slide and gently pipetted to release germ cells. The resulting suspensions were spread onto a new slides containing 1% PFA and 0.15% Triton X-100 (pH 9.2) and incubated in a humidified chamber at 4°C overnight. Following incubation, the slides were washed twice with 0.4% Photo-Flo 200 (Kodak), air-dried at room temperature, and stored at –80°C until further use (28). For subsequent immunofluorescence analysis, the procedure mirrored that of tissue immunofluorescence mentioned earlier.

### Immunoprecipitation and mass spectrometry (co-IP/MS)

Co-IP was conducted using the Co-IP Kit (abs9649, absin) with minor modifications. 80–100 mg of *Hsf5*^+/+^ and *Hsf5*^−/−^ testes were separately dounced with 30 strokes and lysed in 1000 μl of lysis buffer [20 mM Tris–Cl (pH 7.4), 150 mM NaCl, 1% NP-40, 0.25% sodium deoxycholate, 5% glycerol, 1 mM dithiothreitol (DTT) and 1× protease inhibitor], and left on ice for 30 min. After centrifugation at 14 000g, 4°C for 10 min, the supernatant was precleared with protein A/G beads for 2 h before incubation with HSF5 antibodies overnight. Protein A/G beads were then added and rotated for 4 h. The beads were washed five times with cold, high-salt co-IP wash buffer [20 mM Tris–HCl (pH 7.4), 500 mM NaCl, 1 mM EDTA, 5% glycerol, 1% NP-40, 0.25% sodium deoxycholate, 1 mM DTT, with fresh 1× protease inhibitor], rotating each time for 5 min at 4°C. The immunoprecipitated protein complexes were eluted off the beads into sample loading buffer. Elution samples were subjected to MS analyses at Shanghai Bioprofile Technology Co., Ltd (China), and data-dependent acquisition (DDA) mass spectrometry was performed using a Q-Exactive HF-X mass spectrometer (Thermo Scientific).

### Culture of spermatocytes and OA treatment

Short-term culture of testicular cells was conducted following previously reported methods with modifications (29). Approximately 2 × 10^^6^ spermatocytes isolated from postnatal day 18 (P18) *Hsf5*^+/+^ and *Hsf5*^−/−^ mice were cultured overnight in 1 ml of MEMα (A19511, HAKATA) supplemented with 1x penicillin-streptomycin (BC-CE-007, Biochannel), 0.29% dl-lactic acid sodium salt, 0.59% Hepes, and 5% fetal bovine serum in each well of a 6-well plate at 32°C. The following morning, a small fraction of cells (2%) were used to assess cell viability using trypan blue (SNK-004, Sunncell), while the remaining cells were treated with OA (5934S, Cell Signaling Technology) at a final concentration of 4 μM. Cells were harvested 5 h after the addition of OA and utilized for nuclear spread analysis.

### Western blotting (WB)

Briefly, testes or spermatocytes were homogenized in radioimmunoprecipitation assay lysis buffer (P0013B, Beyotime) containing a protease inhibitor cocktail, and the mixture was incubated on ice for 30 min. The lysates were then sonicated for 10 cycles (2 s on/off) at 150W (JY92-IIDN, SCIENTZ) and centrifuged at 14 000g for 10 min at 4°C to remove debris. Subsequently, the lysates were boiled with 5× SDS loading buffer for 10 min. The cell lysates were separated by SDS-PAGE and transferred to polyvinylidene difluoride membranes (88518, Thermo Fisher) using a vertical electrophoresis and blotting apparatus (Bio-Rad). Membranes were blocked in PBST (Phosphate Buffered Saline, 0.3% Tween 20) containing 5% nonfat milk for 30 min and incubated with primary antibodies diluted in PBST buffer containing 5% nonfat milk at 4°C overnight. Following overnight incubation, the membranes were washed with PBST at room temperature with gentle shaking and incubated with horseradish peroxidase (HRP)-conjugated secondary antibodies (A25022, abbkine) for 2 h. Finally, the protein blots were detected using Omni-ECL™ Femto Light Chemiluminescence Kit (SQ201, Epizyme) and imaged on a Bio-Rad ChemiDoc Touch Imaging System. GAPDH (HYK-200184, Shanghai Hengyuan Biological Technology Co., Ltd), α-TUBULIN (FNab00333, FineTest), and Histone 3 (BS1661, Bioworld) were used as loading controls. The other Antibodies used in WB are listed in Supplementary Table S1.

### Plasmid construction and dual luciferase reporter assay

The coding sequence of *Hsf5* was cloned into a pFLAG-CMV-4 vector by Tsingke Biotech (Hangzhou, China). Additionally, the targeted promoter regions of HSF5 for *Sycp1* (+274 bp to –83 bp), *Meiob* (–88 bp to –348 bp), *Hat1* (+75 bp to –237 bp), *Msh4* (–242 bp to –552 bp) and *Tesmin* (+75 bp to –237 bp) were amplified from mouse genomic DNAs, extracted from mouse tail tips, and cloned into a PGL4.23-luciferase vector. HSF5 DBD-expressing plasmids, promoter-luciferase plasmids, and the pRL-TK-Renilla constructs as internal controls were co-transfected into HEK-293T cells (CL-0005, Pricella Life Science & Technology Co., Ltd) cultured in 6-well plates (CAP011006, Jet Biofil) using Lipofectamine™ 3000 reagent (Thermo Fisher), following the manufacturer's protocol. Cell extracts were prepared 36 h post-transfection using the lysis buffer provided in the Dual-Luciferase Reporter Assay System Kit (RG027, Beyotime), and luciferase activity was measured on a Synergy H1 Multi-Mode Microplate Reader instrument (Bio-Tek) according to the manufacturer's protocol. Renilla luciferase activity served to normalize the firefly luciferase activity. The DNA sequences of all targets and primers are listed in Supplementary Table S1.

### EMSA (electrophoretic mobility shift assay)

5′-Cy5 labeled DNAs were synthesized by Tsingke Biotech (Hangzhou, China), with the DNA sequences provided in Supplementary Table S1. Nuclear extracts were obtained from WT spermatocytes purified by STA-PUT method using the Nuclear and Cytoplasmic Protein Extraction Kit (P0028, Beyotime). EMSAs were conducted using the EMSA/Gel-Shift Kit (GS009, Beyotime) following the manufacturer's instructions with minor modifications. For the competitive binding assay, unlabeled probes and labeled mutant probes were introduced into the reaction mixtures at a 100-fold excess compared to the labeled probes and incubated for 20 min before the addition of labeled probes. In super-shift reactions, 2–3 μl of anti-HSF5 antibody was incubated with the reaction mixtures for 20 min at room temperature prior to the addition of labeled DNA probes. All reaction systems were electrophoresed on 5% (w/v) polyacrylamide gels in TBE buffer (45 mM tris borate and 1 mM EDTA, pH 8.3). After electrophoresis, the gel was dried and scanned by Bio-Rad ChemiDoc Touch Imaging System.

### RNA extraction, RT-PCR and qRT-PCR

Total RNA was extracted from various tissues or isolated pachytene spermatocytes using STA-PUT method. SPARKscript II 1st Strand cDNA kit (With gDNA Eraser) (Shandong Sparkjade Biotechnology Co., Ltd.) was employed to reverse transcribe 600 ng of total RNA into cDNA. RT-PCR was conducted using FasHifi Prime DNA Polymerase Mix (Swiss Affinibody LifeScience AG), with 2 μl of 10× diluted cDNA as a template. RT-PCR reactions consisted of an initial denaturation at 98°C for 3 min, followed by 33 cycles of denaturation at 98°C for 30 s, annealing at 60°C for 30 s, and extension at 72°C for 30 s, with a final extension at 72°C for 5 min, using a ProFlex™ PCR system (Thermo Fisher). Subsequently, qRT-PCR was performed using SYBR Green Master Mix (K1070, APExBIO, Houston, USA) with 1 μl of 10× diluted cDNA in a 20 μl reaction mixture in 96-well plates (2041320, SAINING Biotechnology). The PCR reactions were carried out on the CFX96 Touch Real-Time PCR Detection System (Bio-Rad) with the following program: Step 1, 95°C for 2 min for initial denaturation; Step 2, 95°C for 15 s for DNA denaturation; Step 3, 60°C for 30 s for annealing/extension, and Steps 2–3 were repeated for 40 cycles. The relative mRNA levels were quantified using the 2^-ΔΔCt^ method, and all data were normalized to *Gapdh*. Primers used are listed in Supplementary Table S1.

### Cleavage under targets & tagmentation (CUT&Tag) assay

CUT&Tag was conducted following previously described procedures with minor modifications to the CUT & Tag assay (Vazyme, TD904) (30). Approximately 4 × 10^^5^ spermatocytes isolated by STA-PUT method were allowed to bind to ConA beads. The primary antibody and control IgG (CR1, Sino Biological) was incubated with the beads-cell suspension on a rotator overnight at 4°C. The following day, the secondary antibody was added and incubated for 60 min at room temperature, followed by incubation with pA/G-Tn5 transposome and tagmentation in an activating buffer supplemented with magnesium. Adaptor-ligated DNA fragments were extracted and PCR-amplified using indexing primers (TD202, Vazyme). The amplified product was purified with VAHTS DNA Clean Beads (N411-01, Vazyme), and the quantity and quality of the DNA library were assessed using a Qubit™ 4 Fluorometer and Bioanalyzer 2100 (Invitrogen), respectively. The libraries were sequenced on the Illumina sequencing platform by Genedenovo Biotechnology Co., Ltd (Guangzhou, China). Purified DNA was quantified by qRT-PCR, and *Gapdh* (promotor) was used as a reference. The primer sequences used to amplify the predicted binding sites of *Hsf5* are provided in Supplementary Table S1.

### ScRNA-seq library preparation

We performed testis scRNA-seq from P24 *Hsf5*^+/+^ and *Hsf5*^−/−^ mice as previously described (31). Briefly, testes were digested into single cell suspensions as aforementioned method for isolation of spermatogenic cells. Cell concentration was counted by Countess 3 Automated Cell Counter (Thermo Fisher). Mixed the cell suspension with Trypan Blue, and then counted the viability of cells by the introverted microscope. Only samples with > 80% viability were loaded into Single Cell A Chip (10× Genomics, Chromium) and generates single-cell Gel Bead-In-EMlusion (GEMs). libraries were constructed with Single Cell 3′ Reagent Kits v3.1 before sequencing on the Illumina sequencing platform by Genedenovo Biotechnology Co., Ltd (Guangzhou, China).

## Bioinformatics analyses

### Processing of CUT&Tag data

Raw sequence reads were first processed by FastQC (v0.12.1) for quality control, and then poor-quality reads and adapter sequences were removed by cutadapt (v4.6). Quality filtered reads were then mapped to the GRCm39 (mm39) reference genome using Bowtie2 (32) (v2.5.2) with the parameters ‘–local –very-sensitive –no-mixed –no-discordant –phred33 -I 10 -X 700’. Samtools (33) (v1.19.2) was used to calculate read coverage and depth. The peak finding was performed using the factor mode of operation of the findPeaks component in the Homer (34) (v4.11) software package, with default parameters. Subsequently, the peak regions were extensively annotated using the reference annotation dataset (Gencode_M33) with annotatePeaks.pl. Finally, findMotifsGenome.pl was utilized with default parameters to conduct de novo motif discovery within the peak regions. Additionally, DeepTools (35) (v3.5.4) bamCoverage function was used to generate CUT&Tag.

Signal track files in BigWig format. These files were then visualized using IGV (v2.17.3). Peak signals on each chromosome were visualized using ChIPseeker (36) (v1.38). Subsequently, heatmaps of peak signals across all gene bodies were generated using the computeMatrix function and plotHeatmap from DeepTools, along with aligned peak signal heatmaps.

### Processing of 10x genomics scRNA-seq data

Raw sequencing data were converted to fastq format using cellranger (10× Genomics, v7.2.0) mkfastq. scRNA-seq reads were aligned to the GRCm39 (mm39) reference genome and quantified using cellranger count. After collecting digital gene expression count matrix, we performed quality control to the cells based on the distribution of genes detected and UMIs, mitochondrial transcripts percent of each cell for all experiments. The subsequent data processing was performed using the Seurat (37) (v5.0.2) package. We filtered out cells with detected gene counts ≤200 or ≥8500, as well as cells with mitochondrial gene transcript proportions ≥20%. We normalized the raw counts using ‘LogNormalize’ and selected the top 2000 highly variable genes as the basis for dimensionality reduction and clustering. To validate the accuracy of our cell identity annotations, we referenced previously annotated mouse testicular single-cell RNA sequencing data by Chen *et al.* (GSE107644) (38). Subsequently, we employed the anchor-based integration methods provided in Seurat. We defined our samples as the reference samples and used the module activity matrix. We projected cells from the known cell sets into our samples by identifying the mutual nearest neighbor cells for each cell in our samples, thereby assessing whether our annotated cell clusters match with various cell types from the known cell sets. We utilized the MAST (39) to calculate differential genes between single-cell clusters, with a threshold set at |log_2_fold-change| >1 and a P from MAST <0.01 to determine differential expression genes. The gene ontology enrichment analysis of DEGs was conducted on the online website tools DAVID (40). To investigate the inter-cluster correlation among cell populations at the pachytene stage, we employed Seurat's AggregateExpression() function to compute merged pseudo-expression matrices for each cell cluster. We calculated the standard deviation of each gene across cell clusters in the *Hsf5*^+/+^ samples and selected the top 3000 genes with the highest standard deviation, and the Spearman correlation coefficient between the cell clusters of the two samples was calculated.

### RNA velocity and trajectory analysis

To infer the future states of cells and analyze transcriptional dynamics at the level of spliced and unspliced transcripts, we conducted a joint analysis of all transcripts in the single-cell data using Seurat and scVelo. Initially, we utilized cellranger output aligned bam files as input for Velocyto to acquire counts of unspliced and spliced reads in loom format. Subsequently, germ cells and pachytene stage cells were filtered out from Seurat, and their metadata, UMAP dimensional reduction arrays, and clustering information were imported into scVelo (41) (v0.3.1). Each sample loom files were normalized and log transformed using scVelo functions filter_and_normalize(). For the RNA velocity analysis of cells at the pachytene stage, we conducted analysis using scVelo's dynamic modeling. In essence, we used to calculate first and second-order moments for each cell across its nearest neighbors [pp.moments (n_pcs = 30, n_neighbors = 30)]. Then, the recover_dynamics() function was used to restore their dynamic information. Next, the velocities were estimated and the velocity graph was constructed using the tl.velocity() with the mode set to ‘dynamical’ and tl.velocity_graph() functions. Velocities were visualized on top of the previously calculated UMAP coordinates with the tl.velocity_embedding() function. To compute the terminal state likelihood, the function tl.terminal_states() with default parameters was used. The genes with high variability in *Hsf5*^+/+^ pachytene spermatocytes were subjected to maximum likelihood estimation using scvelo's built-in function. All genes for which the calculated ‘fit_likelihood’ > 0 are considered driver genes of the pachytene stage. To assess whether there are changes in the transcriptional dynamics of those genes in *Hsf5*^−/−^, Mann–Kendall tests were conducted on the unspliced mRNA levels of these genes, sorted along the inferred cell time from two samples of pachytene spermatocytes. If the calculated *P* < 0.01 and if the trend changes or the slope ratio obtained by linear regression analysis is greater than 4, it is considered abnormal. Different from the RNA velocity analysis performed by the dynamic modeling of pachytene stage cells, for all germ cells in both samples, we utilized the run_model() function of UniTVelo (42) (v0.2.4), which employs a temporally unified model better suited for longer differentiation paths of cells. The built-in function tl.paga() was used to perform PAGA analysis on the germ cells from Hsf5^−/−^ mice to infer cell differentiation pathways and calculate relationships between cell clusters. All results were visualized using the built-in functions of scVelo.

### Statistical analysis

All experiments reported here were independently repeated at least three times, and all values in the figures are depicted as mean ± s.d. unless stated otherwise. Statistical analyses were conducted using GraphPad Prism 10 software or Excel 2016. Results for two experimental groups were compared by two-tailed unpaired Student's t tests. The levels of significance are indicated as follows: NS, non-significant, *P* < 0.05 (*), *P* < 0.01 (**), *P*< 0.001 (***), and *P* < 0.0001 (****).

## Result

### Meiotic chromatin-associated HSF5 is dominantly expressed through pachynema progression

**Figure 1.**
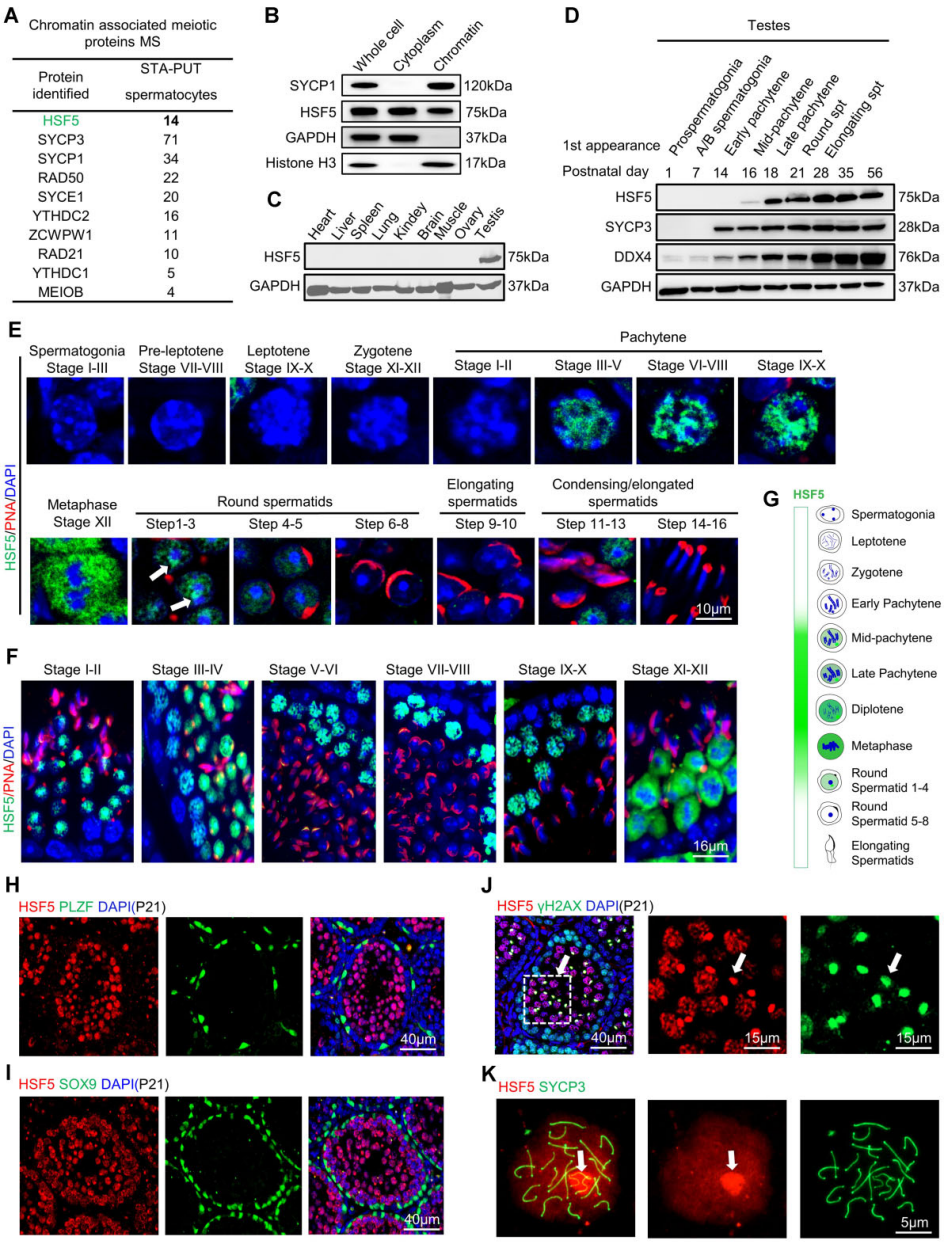
Identification and expression profiles of HSF5 during spermatogenesis in mice. (**A**) Selective chromatin-associated protein candidates identified by MS analysis from spermatocytes purified by STA-PUT method. (**B**) Purity assessment of isolated chromatin-associated proteins and confirmation of the association of HSF5 with chromatin by WB. GAPDH, cytoplasmic positive marker; histone H3, intrinsic component of chromatin; SYCP1, meiotic-specific protein control. (**C**, **D**) WB analyses of HSF5 protein in extracts from P56 mouse tissues and developing mouse testes. Timing of the first appearance of Prespermatogonia, A/B spermatogonia, early pachytene spermatocytes, mid-pachytene spermatocytes, late pachytene spermatocytes, round spermatids (spt), and elongated spermatids in developing testes is shown. SYCP3, meiosis-specific protein control; DDX4, germ cell-specific protein control; GAPDH, loading control. (**E**, **F**) IF analysis of HSF5, γH2AX, and PNA on testes sections at P56. DNA was counterstained with 4′,6-diamidino-2-phenylindole (DAPI). Lower panels show reduction image of seminiferous tubule. Arrow: example of peri-chromocenter area. Scale bar is indicated. (**G**) Graphic representation of developmental expression pattern of HSF5 in male germ cells. (**H**, **I**) PLZF (H) and SOX9 (I) staining with HSF5 on testes sections at P21. Scale bar is indicated. (**J**) γH2AX staining with HSF5 on testes sections at P21. Right two panels show magnification of the boxed areas in the left panel. Arrow: example of XY body. Scale bars are indicated. (**K**) HSF5 staining with SYCP3 on Chromosome spreads of pachytene spermatocytes from P56 *Hsf5*^+/+^ testes. Arrow: example of XY body. Scale bar is indicated.

Previously, we developed a modified method to purify meiotic chromatin-associated proteins (26,43). Using this method, we identified the fifth member of the heat shock factor family (HSF5) as one of the chromatin-associated proteins in spermatocytes (Figure 1A, B and Supplementary Figure S1A–C). Unique peptides of HSF5 were detected in postnatal day 16 (P16) testis samples and exhibited a relatively high peak in spermatocyte samples purified by the STA-PUT method (Figure 1A and Supplementary Figure S1D), which enables the separation of highly pure fractions of spermatocytes from testes by sedimentation at unit gravity through a bovine serum albumin (BSA) gradient (Materials and methods). Multi-alignment and phylogenetic analyses of HSF5 revealed a highly conserved protein encoded by *Hsf5*, expressed in various vertebrate species including mice, humans, rats, horses, rhesus monkeys, etc. (Supplementary Figure S1E and Supplementary Figure S2A, B). Through reverse transcription polymerase chain reaction (RT-PCR) and Quantitative reverse transcription polymerase chain reaction (qRT-PCR), we confirmed exclusive transcripts of *Hsf5* from P16 testis onwards, increasing gradually in developing testes (Supplementary Figure S2C–E), which is consistent with previous scRNA-seq data (31,44).

Subsequently, polyclonal antibodies targeting the HSF5 C-terminal region (amino acids 428–624) were generated (Supplementary Figure S2F). These antibodies were validated for use in Western blotting (WB) and immunofluorescence (IF) analyses (Supplementary Figure S2A, B, G, H). Notably, the WB results identified HSF5 as an approximately 75 kDa protein, which harbors 624 amino acids (predicted molecular mass, 69kDa). HSF5 protein was detected at P16 when mid-pachytene spermatocyte (mP pachynema) first appeared, uniquely present in mice testes (Figure 1C, D). Next, we investigated the cell type specific expression pattern of HSF5 in mouse testis through IF experiments with anti-HSF5, anti-γH2AX (a marker of meiotic DNA damage response), and anti-peanut agglutinin (PNA, an acrosome marker delineating different spermatogenic stages within seminiferous tubules) antibodies (Figure 1E–G). Those IF results revealed predominant nuclear localization of HSF5 in spermatocytes and early spermatids, while it was absent in Sertoli cells and spermatogonia (Figure 1H, I). Detailed analysis indicated detectable HSF5 protein in spermatocytes from stage III seminiferous tubules, reaching peak levels in the late stages of meiotic prophase I, and subsequently declining in postmeiotic spermatids (Figure 1E–G). While HSF5 was concentrated in a peri-chromocenter area in round spermatids characterized by an intermediate DAPI staining intensity (weaker than the chromocenter), it was previously demonstrated to localize to the sex chromosome (45), and eventually disappeared in step 4 spermatids (Figure 1G). Although HSF5 was detected in the cytoplasm (Figure 1B), analysis of spermatocyte nuclear spread and testis sections revealed that HSF5 was diffusely expressed in the nuclei and particularly high signals of HSF5 were seen in the XY body (Figure 1G, J, K). Taken together, HSF5 is a potential meiotic chromatin-associated regulator through pachynema progression, implicating its major function around this timing.

### *Hsf5*-deficient males are infertile with severe meiotic arrest

**Figure 2.**
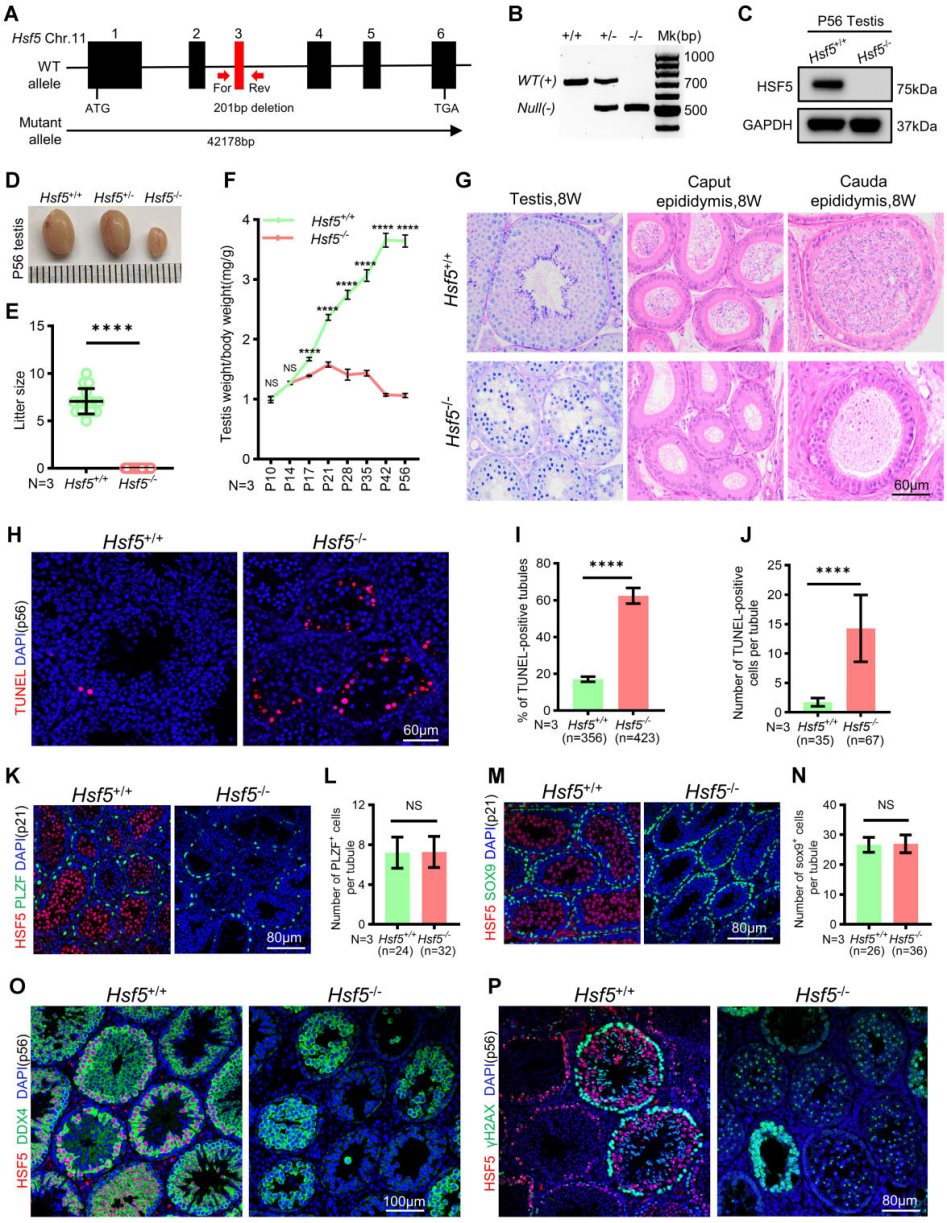
HSF5 is required for male fertility. (**A**) The schematic of *Hsf5* KO allele. Exons 3 were deleted in the mutant. Position of the forward (For) and reverse (Rev) primers used for genotyping are shown. (**B**) *Hsf5* KO was confirmed by PCR (Materials and methods). (**C**) WB confirmation of the elimination of HSF5 protein in *Hsf5*^−/−^ testis. GAPDH, loading control. (**D**) Testes size of P56 *Hsf5*^−/−^ mice were significantly reduced compared to *Hsf5*^+/+^ and *Hsf5*^+/−^ littermates. (**E**) The litter size of adult *Hsf5*^+/+^ males and *Hsf5*^−/−^ males. N, number of males. Data are mean ± s.d. ****P< 0.0001 (Mann–Whitney *U*-test). (**F**) Testes growth curve shows the *Hsf5*^−/−^ testes were significantly decreased from P17. *N*, number of males of each age. Data are mean ± s.d. ****P< 0.0001; NS, non-significant (Two–way ANOVA). (**G**) No spermatids or mature spermatozoa were observed in testis or epididymis by PAS and HE staining in P56 *Hsf5*^−/−^ mice. Scale bar is indicated. (**H**) TUNEL staining on testes sections at P56 from *Hsf5*^+/+^ and *Hsf5*^−/−^ mice; red signals indicate apoptotic cells. Scale bar is indicated. (**I**) Quantitative comparison of TUNEL staining shows that TUNEL-positive tubules were increased in P56 *Hsf5*^−/−^ mice. *N*, number of males; n, number of tubule cross sections analyzed. Data are mean ± s.d. ****P< 0.0001 (unpaired Student's *t*-test). (**J**) TUNEL-positive cells in TUNEL-positive tubules were increased in P56 *Hsf5*^−/−^ mice. *N*, number of males; n, number of TUNEL-positive tubules analyzed. Data are mean ± s.d. ****P< 0.0001 (Mann–Whitney *U*-test). (**K–N**) Immunostaining and quantitative comparison indicated that the number of PLZF and SOX9-positive cells were comparable between P21 *Hsf5*^+/+^ and *Hsf5*^−/−^ mice. Scale bar is indicated. *N*, number of males; *n*, number of tubule cross sections analyzed. Data are mean ± s.d. NS, non-significant (unpaired Student's *t*-test). (**O**, **P**) Immunostaining indicates that DDX4 and γH2AX-positive postmeiotic germ cells were absent in P56 *Hsf5*^−/−^ mice. Scale bar is indicated.

To determine the functional roles of HSF5 in germ cells, we used the CRISPR/Cas9 method to generate a *Hsf5* knockout (KO) mouse model (hereafter also referred to as *Hsf5*^−/−^) by deleting a genomic DNA fragment of 201 bp (base pair) spanning exon 3, which would result in reading frameshifts and premature termination codons (Figure 2A). Genotyping PCR confirmed the absence of full-length transcripts in mice with homozygous mutant alleles, indicating the successful construction of *Hsf5*-deficient mouse model (Figure 2B). WB and IF analyses demonstrated absence of HSF5 protein in testes from adult *Hsf5*^−/−^ mice (Figure 2C; Supplementary Figure S2G, H).

*Hsf5* ^−/−^ mice exhibited viability without discernible developmental anomalies compared to *Hsf5*^+/+^ and *Hsf5*^+/−^ counterparts (‘+’ represents the wild-type (WT) allele). However, *Hsf5*^−/−^ males demonstrated infertility and markedly diminished testicular size relative to *Hsf5*^+/+^ and *Hsf5*^+/−^ littermates (Figure 2D), with fertility tests confirming the infertility of *Hsf5*^−/−^ males but the fertility of *Hsf5*^−/−^ females (Figure 2E and Supplementary Figure S2I). Testicular weights of *Hsf5*^−/−^ mice were substantially reduced across various ages spanning P17 to P56 compared to *Hsf5*^+/+^ (Figure 2F). Histological examination of testes and epididymides in adult *Hsf5*^−/−^ mice revealed severely atrophic seminiferous tubules containing scant germ cells alongside numerous vacuoles, with epididymides devoid of spermatozoa (Figure 2G). Furthermore, frequent observations of apoptotic cells with condensed nuclei were confirmed by terminal deoxynucleotidyl transferase–mediated deoxyuridine triphosphate nick end labeling (TUNEL) assays (Figure 2H). Both the count of TUNEL-positive tubules and TUNEL-positive cells per tubule were significantly elevated in *Hsf5*^−/−^ compared to *Hsf5*^+/+^ testes (Figure 2I, J). Histological evaluation of *Hsf5*^+/+^ and *Hsf5*^−/−^ testes at various developmental stages (P10, 14, 17, 21, 28, 35 and 42) revealed aberrant seminiferous tubules in *Hsf5*^−/−^ testes from P17 onward, characterized by atrophy and the presence of vacuoles (Supplementary Figure S3A). What's more, TUNEL assays indicated a significant increase in apoptotic cells in *Hsf5*^−/−^ testes compared to *Hsf5*^+/+^ counterparts from P17 to P42 (Supplementary Figure S3A, B).

The quantities of undifferentiated spermatogonial stem cells (PLZF^+^) (Figure 2K, L), PCNA (a cell proliferation marker)-positive cells (Supplementary Figure S3C, D), and Sertoli cells (SOX9^+^) (Figure 2M, N) were found to be comparable between *Hsf5*^+/+^ and *Hsf5*^−/−^ mice. These findings implied that there was no striking divergence in the differentiation of spermatogonia and Sertoli cells without *Hsf5*. IF for DDX4 (a germ cell marker) (Figure 2O), γH2AX (Figure 2P), and H1t (a marker for spermatocyte beyond mP stage) (46) (Supplementary Figure S2H) confirmed the absence of postmeiotic germ cells in *Hsf5*^−/−^ testes. To evaluate the integrity of the meiotic process, we assessed meiotic initiation through immunostaining with STRA8 (a marker of differentiating spermatogonia and pre-leptotene spermatocytes). Our observations showed comparable abundance of STRA8^+^ cells in *Hsf5*^−/−^ testes compared to *Hsf5*^+/+^, indicating that the meiotic initiation is unaffected in *Hsf5*^−/−^ testes (Supplementary Figure S3E, F). Collectively, although meiosis is normally initiated and spermatocytes are observed, no postmeiotic germ cells were detected, which indicates that the deletion of *Hsf5* results in meiotic arrest and male sterility, underscoring the essential role of *Hsf5* in meiosis.

### *Hsf5* defect disturbs pachytene exit

**Figure 3.**
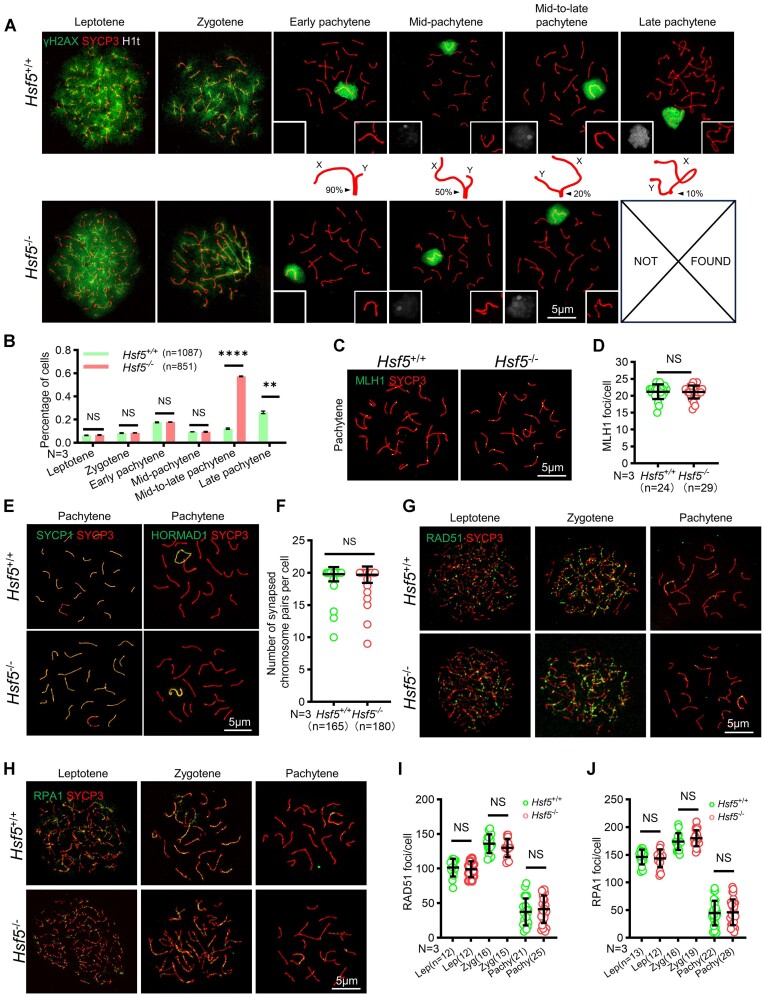
*Hsf5* ^−/−^ disturbs pachytene exit. (**A**) Nuclear spreads of various spermatocytes in P56 *Hsf5*^+/+^ and *Hsf5*^−/−^ mice. Spermatocytes were immunostained with SYCP3, γH2AX, and H1t. H1t signals and magnified XY body are depicted within white boxes embedded in the lower-left and lower-right corners of the corresponding image, respectively. The central panel shows a graphic representation of the XY body, with an arrow indicating the synapsed region of X and Y chromosome. The numbers marked besides the arrows represent the proportion of the synapsed region to the Y chromosome. Scale bar is indicated. (**B**) Frequencies of meiotic stages in P56 *Hsf5*^+/+^ and *Hsf5*^−/−^ spermatocytes. *N*, number of males. Data are mean ± s.d. ****P< 0.0001; **P< 0.01; NS, non-significant (two–way ANOVA). (**C**) MLH1 and SYCP3 immunostaining on chromosome spreads from P56 *Hsf5*^+/+^ and *Hsf5*^−/−^ mice. Scale bar is indicated. (**D**) Scatter plot of MLH1 foci numbers per cell in P56 *Hsf5*^+/+^ and *Hsf5*^−/−^ spermatocytes. *N*, number of males; *n*, number of pachytene spermatocyte analyzed. Data are mean ± s.d. NS, non-significant (Mann–Whitney *U*-test). (**E**) SYCP3 was co-stained with SYCP1 (left two panels) and HORMAD1 (right two panels) respectively on chromosome spreads from P56 *Hsf5*^+/+^ and *Hsf5*^−/−^ mice, Scale bar is indicated. (**F**) Scatter plot of normal synapsed chromosome numbers per cell in P56 *Hsf5*^+/+^ and *Hsf5*^−/−^ spermatocytes. *N*, number of males; *n*, number of pachytene spermatocyte analyzed. Data are mean ± s.d. NS, non-significant (Mann–Whitney *U*-test). (**G**, **H**) SYCP3 was co-stained with RAD5 (G) and RPA1 (H) on chromosome spreads from P56 *Hsf5*^+/+^ and *Hsf5*^−/−^ mice, respectively. Scale bars are indicated. (**I**, **J**) Scatter plot of RAD51 (I) and RPA1 (J) foci numbers per cell in P56 *Hsf5*^+/+^ and *Hsf5*^−/−^ spermatocytes. Lep, leptotene spermatocyte; Zyg, zygotene spermatocyte; Pachy, pachytene spermatocyte. *N*, number of males; *n*, number of spermatocyte analyzed. Data are mean ± s.d. NS, non-significant (one-way ANOVA).

We proceeded to investigate the molecular defects in *Hsf5*^−/−^ spermatocytes by specific markers in meiosis. The substages of meiotic prophase I—leptonema, zygonema, pachynema and diplonema—were delineated through dynamic localization patterns of SYCP3 and γH2AX (28). Pachynema was further subdivided into more specific stages based on chromosomal behaviors: ‘early’, ‘mid-’, ‘mid-to-late’ and ‘late’ pachynema (47) (Figure 3A). Under normal conditions, γH2AX signals in leptonema and zygonema were dispersed throughout the nucleus, indicating numerous unrepaired DSBs. Conversely, in pachytene and diplotene stages, the signals localized to the XY body. The disappearance of γH2AX from pachytene nuclei (except for the XY bodies) suggested repaired autosomal DSBs (48). Late pachytene and diplotene stages were clearly discernible in *Hsf5*^+/+^ through γH2AX and SYCP3 co-staining, while spermatocytes beyond mid-to-late pachynema were absent in the *Hsf5*^−/−^ (Figure 3A). The proportions of leptotene, zygotene, early pachytene (eP), and mP spermatocytes were comparable between *Hsf5*^+/+^ and *Hsf5*^−/−^ mice, while a significant increase in mid-to-late pachytene spermatocytes was noted in *Hsf5*^−/−^ mice (Figure 3A, B). Additionally, no late pachytene spermatocyte was detected among 851 analyzed spermatocytes from *Hsf5*^−/−^ mice, contrasting with normal proportion of late pachytene spermatocytes (26.5%) in *Hsf5*^+/+^ mice (Figure 3B). Regarding MLH1 (a marker for recombination nodule) staining (Figure 3C), we observed an average of 21 MLH1 foci per *Hsf5*^−/−^ spermatocyte, compared to approximately 20 crossover foci per WT one (Figure 3D). Consequently, HSF5 loss led to meiotic arrest at mid-to-late pachytene stages, despite the normal formation of crossovers.

Mid-to-late pachynema arrest can be caused by defects in homologous recombination, synapsis, or meiotic sex chromosome inactivation (MSCI) (49). Initially, we monitored synapsis progression between homologous chromosomes through the co-staining of SYCP3, SYCP1 (a synapsed chromosome marker), and HORMAD1 (an unsynapsed chromosome marker). Compared to *Hsf5*^+/+,^ no aberrant synapsis events were observed in pachytene spermatocytes lacking *Hsf5* (Figure 3E), indicating that *Hsf5*^−/−^ spermatocytes achieve complete synapsis on autosomes and normal pseudo-autosomal region (PAR) synapsis on sex chromosomes. Quantification of synapsed chromosome pairs in *Hsf5*^+/+^ and *Hsf5*^−/−^ testes further supported this observation (Figure 3F). Notably, *Hsf5*^+/+^ testes exhibited 151 cells (91.5%) with fully synapsed chromosome pairs, whereas only 14 cells (8.5%) displayed 10–19 pairs of synapsed chromosomes (Figure 3F). Similarly, in *Hsf5*^−/−^ testes, 163 spermatocytes (90.6%) demonstrated complete synapsis, with 17 cells (9.4%) exhibiting 9–19 pairs of synapsed chromosomes (Figure 3F). Subsequently, we assessed the impact on homologous recombination by quantifying foci of meiotic DSB markers RPA1 and RAD51 across various types of spermatocytes. Our analysis revealed no significant differences in RPA1 and RAD51 foci between *Hsf5*^+/+^ and *Hsf5*^−/−^ (Figure 3G–J). These results indicated that HSF5 was not essential for homologous recombination and synapsis.

**Figure 4.**
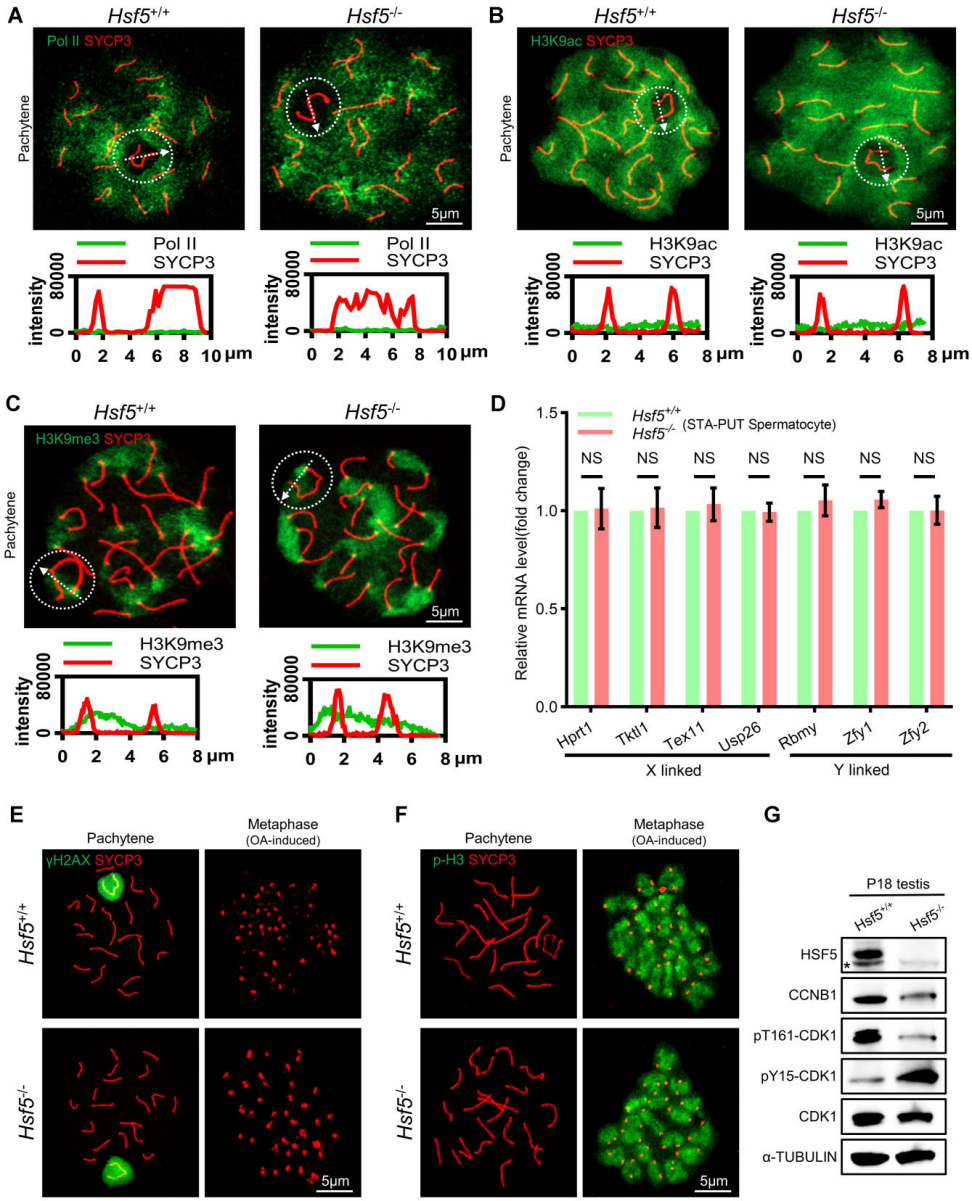
*Hsf5* ^−/−^ pachynema exhibits unaffected MSCI and is competent to Condense Metaphase I Chromosome. (**A–C**) Mid-to-late pachytene spermatocytes from P56 *Hsf5*^+/+^ and *Hsf5*^−/−^ mice were immunostained for SYCP3, Pol II, H3K9ac, and H3K9me3. The correspondingly bottom panel displays plot profiles of the relative fluorescence intensity of these proteins. The dashed circle highlights the XY chromosomes, and arrows indicate the lines used for plot profile analysis. Scale bars are indicated. (**D**) Relative expression levels (fold change) of representative X- and Y-linked genes in spermatocytes isolated with STA-PUT method from *Hsf5*^+/+^ and *Hsf5*^−/−^ determined by qRT-PCR. *Gapdh* mRNA expression levels were used as the loading control. Data are mean ± s.d. and were obtained from three independent experiments. NS, non-significant (two–way ANOVA). (**E**, **F**) Dynamics of γH2AX, SYCP3, p-H3 labeling, and condensation of bivalents were shown in pachytene and metaphase I spermatocytes during the OA-induced G2/MI transition *in vitro*. (**G**) WB of testes lysates from P18 *Hsf5*^+/+^ and *Hsf5*^−/−^ mice with the indicated antibodies. pT161-CDK1, phosphorylation of CDK1 at Thr161; pY15-CDK1, phosphorylation of CDK1 at Tyr15. α-TUBULIN serves as a loading control. Nonspecific bands are labeled with*.

Given HSF5’s predominant localization at the XY body (Figure 1J, K), we hypothesized its involvement in MSCI. To investigate this, we assessed whether HSF5 deletion affected XY chromosome silencing in pachytene spermatocytes (50). Initially, we examined transcriptional activity using RNA polymerase II (Pol II). However, from zygotene to mid-late pachytene spermatocytes, both *Hsf5*^+/+^ and *Hsf5*^−/−^ ones exhibited increased Pol II labeling on the chromatin of all bivalents except sex chromosomes that remained unlabeled through pachynema progression (Figure 4A and Supplementary Figure S4A). Similarly, the signal of histone H3 acetylated at lysine 9 (H3K9ac), which is minimal during zygotene and early pachynema, significantly increased during mid-late pachynema throughout the nucleus but remained low on sex chromosome. H3K9ac displayed a consistent localization pattern in autosomes and sex chromosomes of both *Hsf5*^+/+^ and *Hsf5^−/−^*pachynema (Figure 4B and Supplementary Figure S4B). Histone H3 lysine 9 trimethylation (H3K9me3) is a modification associated with gene silencing and heterochromatin formation. During mid-late pachynema, in both *Hsf5*^+/+^ and *Hsf5*^−/−^, there were obvious declines of H3K9me3 levels observed across autosomes and the X chromosome, with detectable signals at pericentromeric regions and weaker ones around Y chromosomes (Figure 4C and Supplementary Figure S4C) (50). Following this, we assessed the expression levels of XY-linked genes potentially subject to inactivation during meiosis in *Hsf5*^+/+^ and *Hsf5*^−/−^ spermatocytes isolated via STA-PUT method. The qRT-PCR results revealed similar expression levels for the X-linked genes *Hprt1*, *Tktl1*, *Tex11* and *Usp26*, as well as for the Y-linked genes *Zfy1*, *Zfy2* and *Rbmy*, across both groups of spermatocytes (Figure 4D). These collective results implied that HSF5 loss did not impede MSCI despite its predominant localization at the XY body.

### *Hsf5^−/−^* pachynema is competent to condense metaphase I chromosomes

Building upon the aforementioned results, we sought to investigate whether the arrest of *Hsf5*^−/−^ pachynema results from an inability to undergo desynapsis. Exit from meiotic prophase I, or the transition from meiotic prophase I to metaphase I (G2/MI), is initiated by disassembly of the central element of the synaptonemal complex (SC), followed by disassembly of lateral elements of the SC, chromatin condensation and final compaction of distinct MI bivalent chromosomes (29). It is presumed that disassembly and/or reorganization of SC components occur after the completion of meiotic recombination (29). Okadaic acid (OA), a phosphatase inhibitor, induces chromosomal desynapsis in pachynema and triggers entry into cytologically defined metaphase I in WT spermatocytes. As demonstrated earlier, *Hsf5*^−/−^ pachynema exhibited normal meiotic recombination and synapsis; however, no late pachytene spermatocyte or subsequent desynapsis behaviors were observed. Therefore, we attempted to induce cytologically SC disassembly in *Hsf5*^−/−^ pachynema using OA to evaluate their competence for desynapsis. Surprisingly, upon OA treatment, *Hsf5*^−/−^ spermatocytes entered metaphase I, during which SYCP3 labeling disappeared from chromosome arms and accumulated in centromeric regions (Figure 4E). In both *Hsf5*^+/+^ and *Hsf5*^−/−^ spermatocytes treated with OA, γH2AX disappeared, and phosphorylation of histone H3 on Ser10 (p-H3, a marker of chromatin condensation during the diplotene to MI transition), occurred pronouncedly at centromeric heterochromatin in metaphase I (Figure 4F). We next assessed the activity of the maturation promoting factor (MPF) complex, comprising cyclin-dependent kinase 1 (CDK1) and cyclin B1 (CCNB1) which are required to disassemble the SC (51,52). During the exit from meiotic prophase I, CDK1 kinase is activated by binding to CCNB1, phosphorylation at Thr161 (pT161-CDK1), and removal of inhibitory phosphorylation at Tyr15 (pY15-CDK1). Mutants that affect the activation of CDK1 cause SC-disassembly defects and arrest in the mid- to late pachytene (11,53). In *Hsf5*^−/−^ testes, there was a significant decrease in pT161-CDK1 and CCNB1 levels compared to *Hsf5*^+/+^, alongside a notable increase in pY15-CDK1 (Figure 4G), indicating a substantial impairment in MPF activity which may lead to *Hsf5*^−/−^ pachynema failing to exit meiotic prophase I. Above findings indicated a requirement for HSF5 in the desynapsis and disassembly of the SC, thus facilitating the exit from meiotic prophase I in male meiosis.

### HSF5 preferentially binds to promoter regions of genes with a unique target specificity

**Figure 5.**
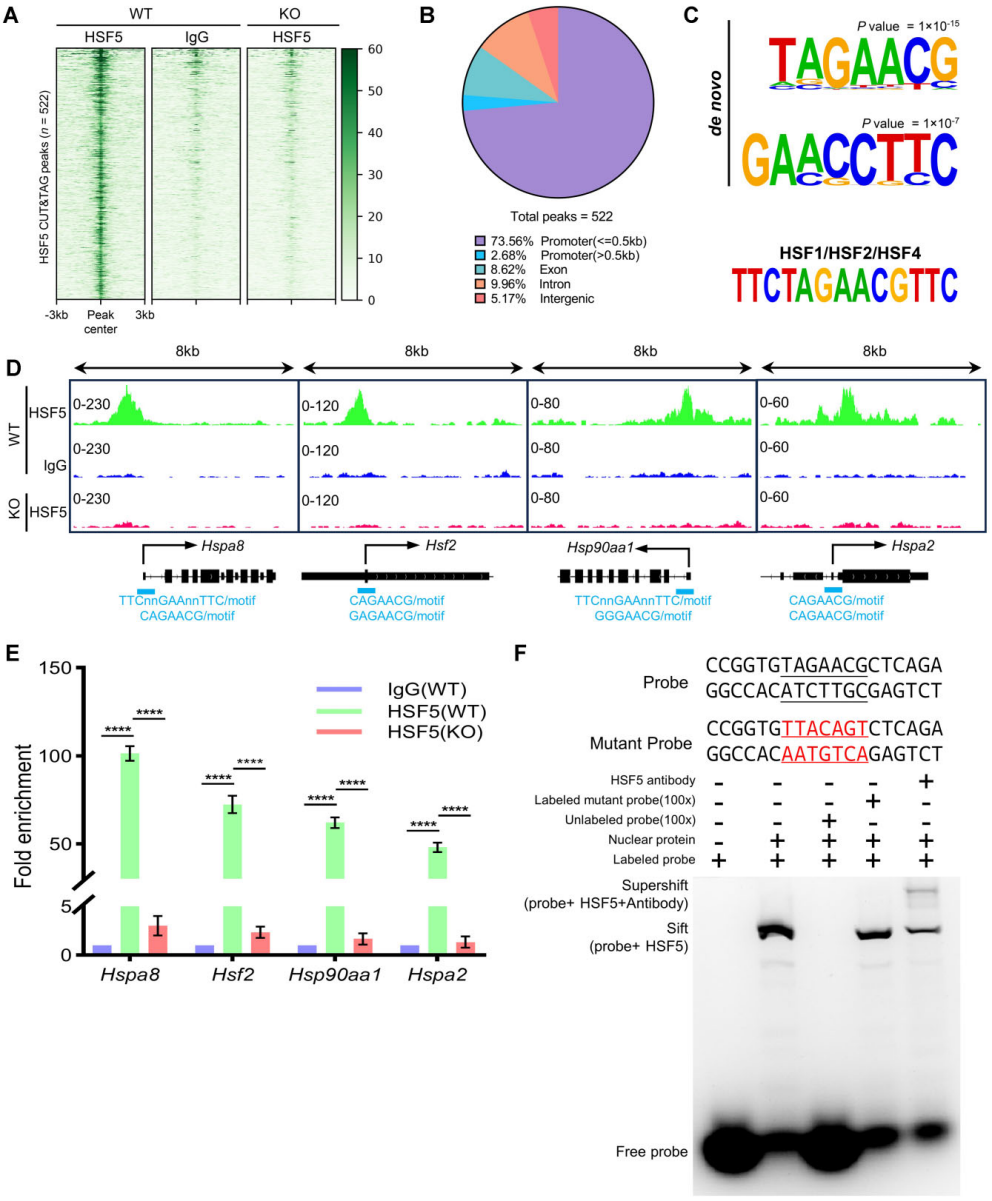
HSF5 binds predominantly to the promoters of multiple pachytene expressed genes with a unique target specificity. (**A**) Heatmap of HSF5 CUT&Tag enrichment across all peaks (*n* = 522) in the mouse genome. Each row represents a 6-kb window centered on HSF5 peak midpoints, sorted by the HSF5 CUT&Tag signal. IgG signals and KO signals at the same position are shown on the right. (**B**) HSF5-binding sites were classified by their genomic locations as indicated. (**C**) Top *Hsf5* DNA binding motif predicted de novo by HOMER (top panel). The motif shares sequence characteristics similar to those of known HSF1/HSF2/HSF4 motifs (bottom panel). (**D**) The track plot of CUT&Tag signals around the TSS of select heat shock family genes to HSF5, with the positions of HSE and certain motifs marked at their genomic locations. (**E**) CUT&Tag-qPCR verified the enrichments of HSF5 at the promoters of *Hspa8*, *Hsf2*, *Hsp90aa1*, and *Hspa2* in *Hsf5*^+/+^ and *Hsf5*^−/−^ pachytene spermatocyte. *Gapdh* promoter was used as a negative control. Data are mean ± s.d. and were obtained from three independent experiments. ****P< 0.0001 (two-way ANOVA). (**F**) The DNA binding ability of the HSF5 was evaluated using an EMSA assay. Displayed at the top are the sequences of the target probe and mutant probes. The target probe DNA is from the promoter fragment of *Slc25a42* (+189 bp to +207 bp), which is highly bound by HSF5 (Supplementary Figure S5C, D) and contains a heptamer motif (TAGAACG) in its promoter region, as depicted in (C).

Given HSF5’s nuclear localization and possession of a winged-helix-turnhelix (WHTH)-like DNA-binding domain (18) (Supplementary Figure S2F), we hypothesize that HSF5 achieves its function by specifically binding to target sites on the genome. To characterize HSF5-binding sites, CUT&Tag analyses were performed separately using purified spermatocytes from *Hsf5*^+/+^ and *Hsf5*^−/−^ mice, identifying a total of 522 peaks (Figure 5A; Supplementary Figure S5A, B; Supplementary Table S2), with validation of the top 3 peak genes (*Cdca2*, *Vcf1*, and *Slc25a42*) conducted via CUT&Tag-qPCR (Supplementary Figure S5C, D). Significantly, HSF5 peaks showed enrichment in proximal promoters (from –50 0b to +500 bp of transcriptional start sites, 73.56%), promoters (from >500 bp of transcriptional start sites, 2.68%), exons (8.62%), introns (9.96%) and intergenic regions (5.17%) (Figure 5B; Supplementary Figure S5A). Further investigation of the enrichment of HSF5 peaks with known or novel motifs unveiled that the HSF5-binding motif consists of the heptamer (T/A/C) (A/G)GAA (C/T) (G/C/A) (*P* = 1 × 10^–15^) and octamer GA (A/C) (C/G)C (T/G) (T/C)C (*P* = 1 × 10^–7^), with a pronounced preference for a single triplet GAA (Figure 5C). Additionally, HSF5 was found to recognize a typical heat shock element (HSE) consensus sequence, consisting of a tandem array of at least three oppositely oriented ‘nGAAn’ motifs or a degenerate version thereof, bound by other canonical HSFs family transcription factors (Figure 5C) (54). Besides its preferential binding to the heat shock proteins (HSPs) promoter regions, such as *Hspa8*, *Hsp90aa1* and *Hspa2*, HSF5 also demonstrated high affinity towards the promoter region of heat shock transcription factor family 2 (*Hsf2*), suggesting that *Hsf5* could likely function as an epistatic master regulator in the HSFs family interaction network in addition to its canonical role in controlling HSP transcription, as confirmed by CUT&Tag-qPCR analysis (Figure 5D, E). Furthermore, electrophoretic mobility shift assay (EMSA) was performed to assess their binding affinity to the heptamer motif (TAGAACG) predicted from CUT&Tag analyses (Figure 5C). The results indicated that motif probes preferentially bound to nuclear extracts, with the binding signal becoming undetectable in the presence of an excess amount of unlabeled DNA containing the target motif but not the mutant sequence, thus demonstrating the DNA-binding specificity of HSF5 to the predicted motif (Figure 5F). In summary, these findings suggested that besides HSE, HSF5 may preferentially bound to a motif consists of the heptamer (T/A/C) (A/G)GAA (C/T) (G/C/A).

### *Hsf5* deletion leads to misled spermatocyte progression trajectory

**Figure 6.**
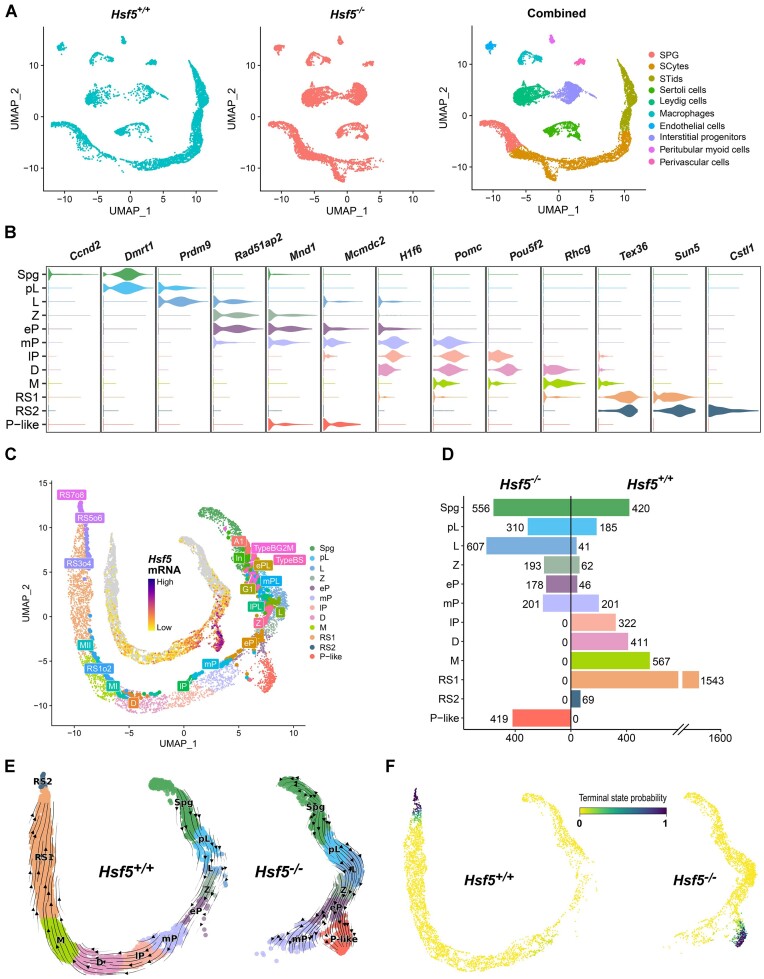
Overview of single-cell transcriptome profiling on P24 *Hsf5*^+/+^ and *Hsf5*^−/−^ whole testes. (**A**) UMAP and clustering analysis on the single-cell transcriptomes of testes from *Hsf5*^+/+^ (*n* = 5677 cells) and *Hsf5*^−/−^ (*n* = 6532 cells) mice. The UMAP dimensional reduction results of single cells from both samples are presented separately (left panel one and left panel two). Each dot in the combined results represents a single cell and is colored based on its cluster identity as indicated in the figure legend (right panel one). UMAP, Uniform Manifold Approximation and Projection; SPG, roughly identified spermatogonia; SCytes, meiotic spermatocytes; STids, post-meiotic haploid round spermatids. (**B**) Violin plots showing the stage-specific representative gene expression based on combined results of single-cell transcriptomes throughout spermatogenesis stages. The width of the violin represents the expression density of the gene within the cell population, while the length of the line depicts the distribution of expression levels, with outliers unmarked. Scales of each gene expression are independent of each other. (**C**) Isolate all germ cells (n_*Hsf5*^+/+^ = 3867, n_*Hsf5*^−/−^ = 2464) and perform re-UMAP and clustering analysis. Each dot represents a single cell and is colored according to its cluster identity as indicated in the figure legend (Spg, spermatogonia; pL, preleptotene; L, leptotene; Z, zygotene; eP, early pachytene; mP, middle pachytene; lP, late pachytene; D, diplotene; M, metaphase; RS1, steps 1–4 spermatids; RS2, steps 5–7 spermatids; P-like, pachytene-like). Furthermore, map germ cells from a reference dataset with known cell identity onto the current UMAP space to validate cell clustering and annotation. Cells from the reference dataset colored according to their identity, and their identities are labeled accordingly. The reference dataset features more detailed annotations of cell types (A1, type A1 spermatogonia; In, intermediate spermatogonia; BS, S phase type B spermatogonia; BG2, G2/M phase type B spermatogonia; G1, G1 phase preleptotene; ePL, early S phase preleptotene; mPL, middle S phase preleptotene; lPL, late S phase preleptotene; MI, metaphase I; M II, metaphase II; RS1o2, steps 1–2 spermatids; RS3o4, steps 3–4 spermatids; RS5o6, steps 5–6 spermatids; RS7o8, steps 7–8 spermatids). (**D**) The symmetrical bar plot displays the quantities of various types of cells from two samples, with the cell counts labeled on the graph. (**E**) Visualization of the RNA velocity analysis results on the UMAP plot. The prediction of future cell states, displayed as streamlines, reveals the overall trend of spermatogenic cell differentiation in each sample at the single-cell level. The thickness and density of the arrows represent the intensity of the cell differentiation trend indicated by the velocity vector field along the direction of the arrows. Compared to the linear differentiation pathway in *Hsf5*^+/+^ cells, the differentiation pathway in *Hsf5*^−/−^ cells exhibits branching and ends prematurely. (**F**) Predicted terminal points based on velocity directed trajectory (E) and colored the cells according to their differentiation terminal probability.

To investigate the impact of HSF5 on gene expression at a cell-type resolution, we conducted scRNA-seq analysis on testicular cells obtained from *Hsf5*^+/+^ and *Hsf5*^−/−^ mice at P24, a stage where spermatids approximately reach steps 5–7 during the first wave of spermatogenesis in *Hsf5*^+/+^, consistent with the *Hsf5* expression pattern (Figure 1G). After filtering out low-quality cells, 5677 *Hsf5*^+/+^ and 6532 *Hsf5*^−/−^ single cells were retained for further analysis. To evaluate both the compositional changes in cell types and differences in transcriptional regulation, we independently normalized the single-cell expression profiles of the two samples in separate layers and then clustered them using the same parameters and removed batch effects in UMAP analysis (Figure 6A). Utilizing previously described germ cell-specific markers (44,55), we identified spermatogenic cell clusters including: spermatogonia (SPG), meiotic spermatocytes (SCytes), and post-meiotic haploid round spermatids (STids) (Figure 6A). Additionally, seven somatic cell compartments were identified, including Sertoli, Leydig, macrophage, endothelial, interstitial progenitor, peritubular myoid, and perivascular cells (Figure 6A), characterized by specific markers (Supplementary Figure S6A, B) consistent with previous studies (56). Furthermore, considering that HSF5 is primarily expressed in germ cells and somatic cells are not affected in HSF5-deficient testes (Figure 1E and Figure 2M), we zoomed in germ cell for re-clustering to get more specific and detailed cell cluster, and we identified 12 subtypes (Figure 6B). To validate our germ cell type identification, we compared our results with previous fluorescence-activated cell sorting (FACS)-based smart-seq2 scRNA-seq data (38), demonstrating consistency in cell type assignments, with HSF5 expression pattern basically coinciding with our IF results (Figure 1E-G, Figure 6C). Notably, further analysis of the cell number within each germ cell sub-cluster indicates that the proportion of spermatocytes beyond the mP stage was completely absent (Figure 6D), confirming our aforementioned findings (Figure 3A, B). Remarkably, *Hsf5*^−/−^ mice exhibited a unique cluster with a transcriptome distinct from other pachytene spermatocyte clusters, labeled as ‘Pachytene-like’ (P-like). This P-like cluster expressed spermatocyte markers *Mind1* and *Mcmdc2*, but not *Rad51ap2*, *H1f6*, *Pomc*, and *Pou5f2*, while exhibiting high specificity in expression of a group of genes including *Cenpp*, *Gab2*, and *Nbea* whose function in spermatogenesis are unclear (Figure 6B, Supplementary Table S3). This marked reduction in late pachytene spermatocytes and the presence of P-like spermatocytes with abnormal transcriptomes in *Hsf5*^−/−^ testes emphasized the crucial role of HSF5 in the pachynema progression.

Given that scRNA-seq is instrumental in elucidating abnormal trajectory events in perturbed samples, which lead to deviations from normal cell states, we performed RNA velocity analysis based on single-cell splicing kinetics using the scVelo analysis toolkit (57). RNA velocity is a high-dimensional vector that can predict the future state of individual cells, thereby presenting the directional differentiation trend of spermatogenic cells at the single-cell level. Unlike the WT germ cells, which exhibited a robust velocity pattern stemming from a proliferating progenitor spermatogonia state and progressing through a sequence of intermediate spermatocyte stages to more mature differentiated spermatids (Figure 6E), early *Hsf5*^−/−^ pachynema partially advanced towards mP but predominantly deviated towards a P-like state, characterized by significantly reduced RNA velocity as indicated by the majority of arrows (Figure 6E). We observed a significant slowdown in the RNA velocity of P-like cells in the stream plot of velocities, indicating that the differentiation process of P-like cells was likely to have become stagnant (Figure 6E). The Markov random walk model can be used to study cell state transitions, where each cell state is considered a node and transition probabilities between nodes are determined by the RNA velocity field (57). Cells with a very low probability of transitioning to other nodes are predicted to represent the terminal state in one sample. Of note, the predicted terminal state does not directly signify a physiological end-stage of the cell, but rather indicates the final stage of differentiation in a given population of cells. RS2 (steps 5–7 spermatids) has the highest terminal state probability among spermatogenic cells in *Hsf5*^+/+^, which is consistent with the fact that spermatids approximately reach the steps 5–7 during the first wave of spermatogenesis at P24, while the most likely terminal state in *Hsf5*^−/−^ spermatogenic cells is predicted to be P-like (Figure 6F). This suggests that P-like is the final state *Hsf5*^−/−^ spermatogenic cells can reach in the spermatogenesis differentiation pathway. Besides, the results of velocity pseudotime analysis also showed that P-like spermatocytes were located at the end of the germ cells differentiation pathway in *Hsf5*^−/−^ mice (Supplementary Figure S6C). Taken together, these analyses demonstrated that *Hsf5* deficiency altered the differentiation and progression of pachynema.

### *Hsf5* deletion disrupts the transcriptomes of spermatocytes in pachynema progression

**Figure 7.**
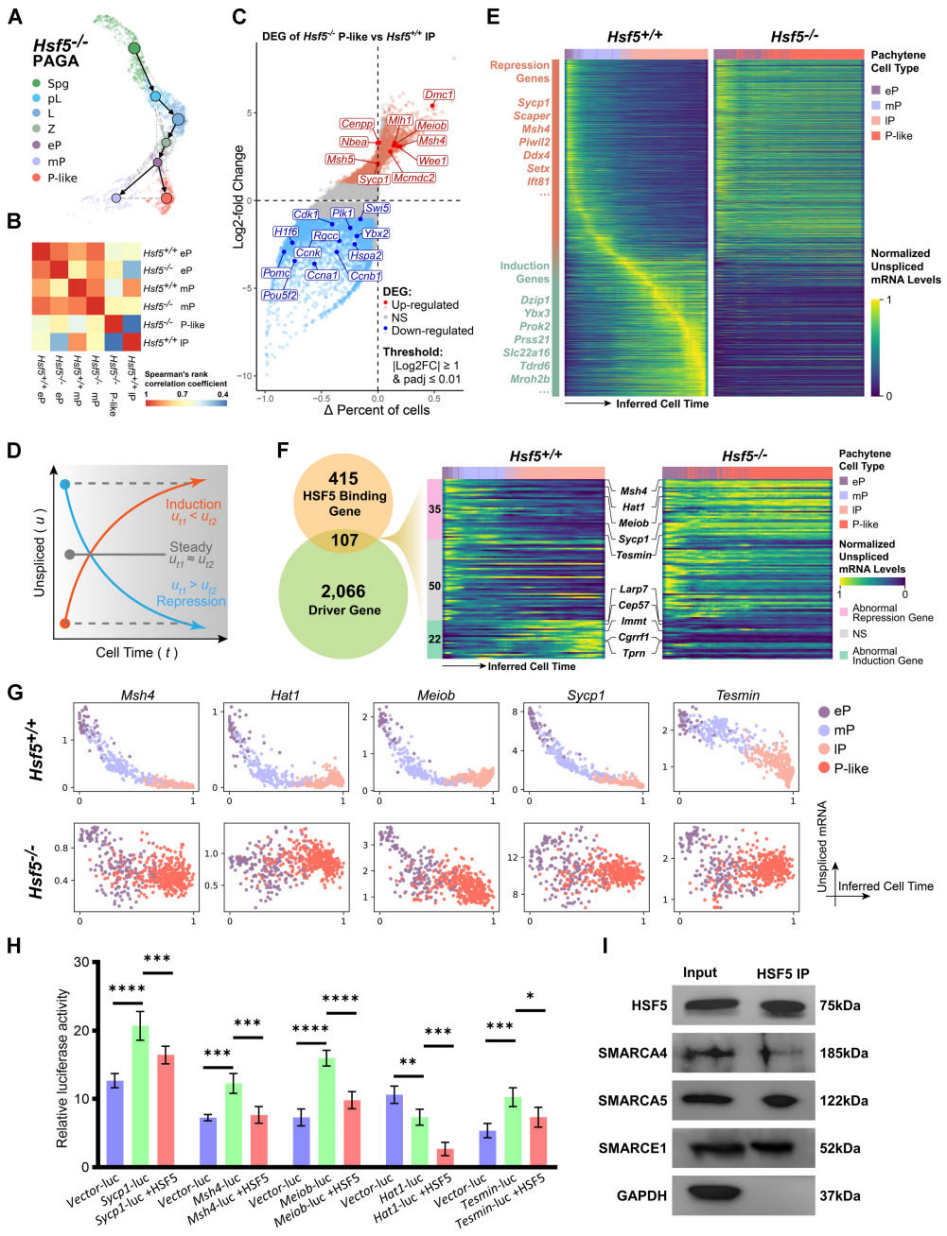
The gene expression of spermatocytes and the transcriptional dynamics of pachynema progression driver genes are compromised following *Hsf5* KO. (**A**) The Partition-based graph abstraction (PAGA) plot depicts the differentiation trajectory of *Hsf5*^−/−^ germ cells, with solid arrows indicating the predicted cell differentiation trajectories, and gray dashed lines indicating the similarity between cell clusters. Thicker gray dashed lines represent higher similarity between clusters. (**B**) The heatmap of cell type gene expression Spearman correlation between our annotated pachytene stage germ cells reference expression profile. The color bar denotes the correlation coefficients between each annotated cell type. (**C**) Differential gene expression analysis using the log_2_fold-change expression versus the difference in the percentage of cells expressing the gene comparing *Hsf5*^−/−^ P-like cells versus *Hsf5*^+/+^ lP cells (Δ percentage difference). The threshold for differential genes is log_2_fold-change > 1 and adjusted P-value from model-based analysis of single-cell transcriptomics (MAST) < 0.01. (**D**) According to the trend of transcription dynamics in inferred cell time, driver genes can be divided into repression genes and induction genes. (**E**) The heatmap of gene transcriptional dynamics resolved along inferred cell time shows clear transcriptional cascades for pachytene driver genes (*n* = 2173) from *Hsf5*^+/+^ (left panel), as well as the transcriptional dynamics of these genes in *Hsf5*^−/−^ (right panel). Each row represents a gene, and each column represents a single cell, with columns/cells placed by inferred cell time order obtained from RNA velocity analysis and depicted by a thick colored line based on its cluster identity as indicated in the figure legend. Compared to levels of spliced mRNA or total mRNA, levels of unspliced mRNA better reflect the transcription dynamics of genes. Unspliced mRNA levels for each individual gene were normalized uniformly across the two samples. (**F**) The numbers of driver genes and HSF5 binding genes, as well as their overlapping gene (left panel). Mann–Kendall test is conducted on the unspliced mRNA levels of cells sorted along inferred cell time, comparing the transcriptional trends of overlapping genes between*Hsf5*^+/+^ and *Hsf5*^−/−^. These 107 genes are classified into three categories: repression trend anomaly (*n* = 35), induction trend anomaly (*n* = 22), and trend unchanged (no change or *P*-value > 0.01, *n* = 50). Transcriptional dynamics of these HSF5 binding driver genes were visualized by heatmaps of unspliced mRNA along inferred cell time (right panel). (**G**) The changes in unspliced mRNA levels of driver genes binding by HSF5 at single-cell resolution along the inferred cell time. Each dot represents a single cell and is colored according to its cluster identity. The values along the x-axis and y-axis represent the order of cells along the inferred cell time and the unspliced mRNA levels of the gene, respectively. (**H**) Validation of DNA binding motif of HSF5 using dual-luciferase assay. Data are mean ± s.d. and were obtained from three independent experiments. ****P< 0.0001 (two-way ANOVA). (**I**) Co-IP of HSF5 with SMARCA5, SMARCA4 and SMARCE1 in testis from WT. GAPDH, loading control.

In our preceding analysis, we observed that the majority of cells with a high probability of terminal states in *Hsf5*^−/−^ are clustered within the P-like cluster, suggesting that P-like cells are likely the final stage of spermatogenesis that can be reached in *Hsf5*^−/−^ mice. Therefore, we endeavored to elucidate the origin and trajectory of the P-like cluster upon *Hsf5* depletion. Partition-based graph abstraction (PAGA) is a graph-based analytical method that constructs a directed topological map by integrating information from a k-nearest neighbor (kNN) graph and RNA velocity at the single-cell level, revealing potential relationships and developmental trajectories between cell clusters (58). Using PAGA to analyze associations and differentiation pathways between cell clusters in *Hsf5*^−/−^, we found that P-like and mP were two branches derived from eP (Figure 7A). Based on the above series of analysis results, we speculate that although a small number of eP cells could enter the mP stage, they eventually stagnated in the P-like state (Figure 7A), which also explained the numerical dominance of P-like ones among the pachytene stage cells in *Hsf5*^−/−^ mice (Figure 6D). In conclusion, P-like cells with abnormal expression patterns differentiate from eP cells in *Hsf5*^−/−^ mice, and meiotic progression in *Hsf5*^−/−^ mice eventually arrests at this stage.

Subsequently, we concentrated on spermatocytes (re-UMAP) for further analysis to elucidate the gene expression dynamics across these four clusters (eP, mP, lP and P-like) (Supplementary Figure S7A), uncovering distinct differences in transcriptomes between *Hsf5*^+/+^ and *Hsf5*^−/−^ samples (Figure 7B; Supplementary Table S3). We analyzed the DEGs of these four clusters of spermatocytes and found significant enrichment in GO terms ‘spermatogenesis,’ ‘DNA repair,’ ‘meiotic cell cycle’, ‘cell division’ and ‘cell differentiation’ (Supplementary Figure S7B). The DEGs exhibited dynamic, cluster-specific expression patterns during WT pachynema progression and displayed aberrant expression in P-like cells, with high expression in WT pachynema corresponding to low expression in P-like cells, and vice versa (Supplementary Figure S7B), suggesting their crucial role in advancing pachynema progression. To be specific, we performed pairwise comparisons of these cell clusters. Compared to *Hsf5*^−/−^ and *Hsf5*^+/+^ eP respectively, we identified 822 up-regulated and 578 down-regulated DEGs in *Hsf5*^−/−^ P-like, as well as 416 up-regulated and 380 down-regulated DEGs in *Hsf5*^+/+^ mP. Notably, 19.3% (159 of 822) of the up-regulated DEGs in P-like and 38.2% (159 of 416) of the up-regulated DEGs in mP overlapped with each other, while 11.9% (69 of 578) of the down-regulated DEGs in P-like and 18.2% (69 of 380) of the down-regulated DEGs in mP also exhibited overlap (Supplementary Figure S7C; Supplementary Table S3). The analysis of DEGs between mP and P-like identified numerous previously reported spermatogenesis indispensable genes such as *Dmc1*, *Wee1*, *Hsf2*, *Hspa2* etc., and the top 3 Gene Ontology (GO) terms enriched by these DEGs were ‘Cellular response to DNA damage stimulus’, ‘DNA repair’ and ‘Cell cycle’ for P-like-upregulated, and ‘Translation’, ‘Spermatogenesis’ and ‘Flagellated sperm motility’ for P-like-downregulated (Supplementary Figure S7D, E). Together, these data indicated that dysregulated expression of these DEGs results in P-like and mP cells, though originating from eP, following different differentiation trajectories and possessing distinct transcriptomes. Next, we analyzed the DEGs between P-like and lP to explore the reasons behind P-like clusters' differentiation arrest with a terminate state. We observed a significant reduction in genes crucial for SC disassembly and G2/M transition, such as *Hspa2*, *Cdk1*, *Ccnb1*, *Ccna1* and *Plk1*, in P-like cluster (Figure 7C; Supplementary Table S3). Conversely, pachytene checkpoint-associated genes, including *Wee1*, *Dmc1*, *Sycp1* and *Msh5*, showed notable upregulation. Combining with our previous findings of impaired SC disassembly (Figure 4E), reduced MPF activity (Figure 4G), and increased spermatocyte apoptosis (Figure 2H–J) in *Hsf5*^−/−^ mice, these results suggested that *Hsf5* depletion disrupts SC desynapsis initiation and activates pachytene checkpoint mechanisms, ultimately leading to P-like spermatocytes apoptosis.

### HSF5 orchestrates the transcriptional dynamics of driver genes in pachynema progression

The analysis of DEGs offers insights into how the absence of *Hsf5* disrupts the transcriptome of pachytene spermatocytes. However, this approach merely captured a static snapshot of the transcriptome of a cell population at a given moment, thus lacking a time-resolved interpretation of transcriptional dynamics. Considering that HSF5 is predicted to function as a transcription factor (Figure 5A), we used a toolkit to estimate RNA velocity by leveraging splicing kinetics, to elucidate HSF5’s regulation on transcriptional dynamics and its role in driving pachynema progression. ScVelo (41), which collects different methods for inferring RNA velocity using an expectation-maximization framework, deep generative modeling, or metabolically labeled transcripts (Figure 7D), was applied. Briefly, genes are classified into three categories based on their transcription dynamics during development: ‘steady genes’ maintain constant unspliced (nascent) mRNA abundance, ‘induction genes’ show increasing unspliced mRNA abundance, and ‘repression genes’ show decreasing unspliced mRNA abundance. The balance among these gene categories drives cellular progression and indicates the cell's future state and fate. Using this toolkit, as spermatocytes progressed from eP to lP in *Hsf5*^+/+^ mice, we identified a set of repressed genes including *Sycp1*, *Scaper*, *Msh4*, *Piwil2*, *Ddx4*, *Setx*, *Ift81* etc., and an induced genes set comprising *Dzip1*, *Ybx3*, *Prok2*, *Prss21*, *Slc22a16*, *Tdrd6*, *Mroh2b* etc. (Figure 7E). These identified repression and induction genes exhibit dynamic changes consistent with their reported function in spermatocyte progression (59–62), indicating the establishment of a high-confidence driver genes reference manifold based on our *Hsf5*^+/+^ pachynema, onto which *Hsf5*^−/−^ pachynema were projected. In the *Hsf5*^+/+^ pachynema, induction genes are sequentially activated and repression genes are gradually turned off in an orderly manner to prevent conflicts between previous and ongoing biological processes. However, these ordered changes in the transcriptome cease in *Hsf5*^−/−^ pachynema, lacking the dynamic progression observed in *Hsf5*^+/+^ pachynema. To be specific, in *Hsf5*^−/−^ pachynema, repression genes maintained moderately continuous high transcription as inferred cell time progressed, while induction genes appeared to fail in transcription increasement (Supplementary Figure S8A). These findings suggested that the disorder in the dynamics of pachynema progression driver genes contributed to spermatocyte arrest upon *Hsf5* deletion.

To further decipher whether HSF5 regulates these pachynema progression driver genes directly, we overlapped HSF5-bound targets (Figure 5A) with the driver genes and obtained 107 HSF5-bound driver gene targets (DGTs) (Figure 7F; Supplementary Table S4). Subsequently, we compared the transcriptional dynamics of these 107 HSF5-bound DGTs between *Hsf5*^+/+^ and *Hsf5*^−/−^ pachynema. Intriguingly, HSF5-bound DGTs displayed significant dysregulation in *Hsf5*^−/−^ pachynema (Figure 7F), with 32.7% (35 of 107) of them showing sustained high transcription levels without decline (Figure 7F), and 20.6% (22 of 107) of them maintaining low transcription levels without increase over the inferred cell time (Figure 7F). Further analysis of these HSF5-bound DGTs with abnormal transcriptional dynamics in *Hsf5*^−/−^ pachynema revealed that the aberration in the transcriptional dynamics of repression driver genes was particularly prominent, including genes such as *Sycp1*, *Msh4*, *Tesmin*, *Meiob*, *Hat1* etc. (Figure 7G); whereas the transcriptional dynamics of several induction driver genes were also mildly altered (Supplementary Figure S8B). However, since the 22 HSF5-bound induction genes drive lP progression (Figure 7E, F), and no spermatocytes progress to the lP stage in *Hsf5*^−/−^ mice, it is unclear if the altered transcriptional dynamics are due to *Hsf5* deficiency or the absence of lP spermatocytes. Therefore, our conclusions focus on the possibility that HSF5 may repress certain target genes during pachynema progression, though it might also promote some genes during this process.

To verify this hypothesis, we performed dual luciferase assays to examine whether HSF5 suppressed the expression of repression driver genes by binding to genomic regions identified through CUT&Tag-seq analyses (Figure 5B and Figure 7F, G). HSF5 significantly reduced the activities of the five binding sites (*Sycp1*, *Msh4*, *Tesmin*, *Meiob* and *Hat1*), irrespective of whether these sites augmented or attenuated the activity of the basal promoter in the luciferase plasmid (Figure 7H). Furthermore, to investigate the involvement of HSF5 in transcriptional regulation and its potential interactions with other factors, we conducted immunoprecipitation mass spectrometry (co-IP/MS) using anti-HSF5 antibodies. The co-IP/MS results demonstrated the immunoprecipitation of SMARCA5, SMARCA4 and SMARCE1 with HSF5 (Supplementary Table S5), which were further confirmed by WB (Figure 7I). Considering that SMARCA5, SMARCA4 and SMARCE1 are subunits of the ISWI and SWI/SNF complexes which can repress transcription *in vivo* through their nucleosome remodeling activity, it was plausible that HSF5 may collaborate with these complexes to negatively regulate the expression of certain HSF5-bound genes (63–65). Meanwhile, several published papers may support this hypothesis. For example, it's reported that females with oocyte-specific depletion of SMARCA5 are infertile and oocytes lacking SMARCA5 fail to undergo meiotic resumption, and their RNA sequencing analysis of growing oocytes from early secondary follicles, secondary follicle as well as fully grown oocytes from preovulatory follicles showed down-regulated expression of HSF5-regulated genes *Msh4*, *Meiob* and *Sycp1* (66). Abnormal *Sycp1* expression pattern and synapsis are impaired in the absence of SMARCA4 (67). Additionally, SMARCE1 is involved in gene transcription repression in non-small cell lung cancers (NSCLCs) (68). Collectively, these results support that, by interacting with SMARCA5, SMARCA4, and SMARCE1, HSF5 may act as a transcriptional suppressor by binding to the promoters of certain driver genes for pachynema progression.

**Figure 8.**
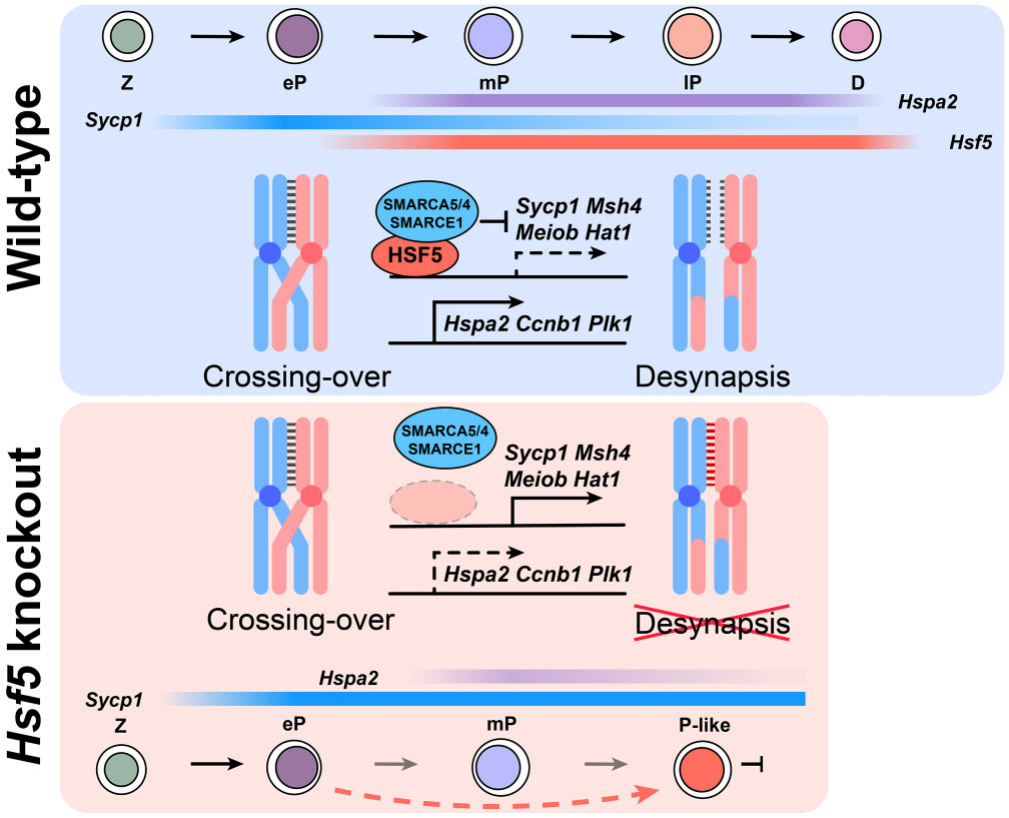
Schematic diagram illustrating the proposed role of HSF5 in pachynema progression during male meiosis. Following the eP stage, genes such as *Sycp1*, *Meiob*, *Msh4*, and *Hat1* are gradually downregulated, while genes like *Hspa2*, *Ccnb1*, and *Plk1* are upregulated from mid- to late pachytene (mP to lP) to facilitate the next step of desynapsis. In the absence of HSF5, genes that should be downregulated continue to be transcribed at high levels, whereas genes that should be upregulated remain at low transcription levels. This dysregulation of gene expression ultimately leads to the misled spermatocyte progression trajectory, failure of pachytene exit, and meiotic arrest (eP, early pachytene; mP, middle pachytene; lP, late pachytene; D, diplotene; P-like, pachytene-like).

In summary, based on the results, we propose a model for the function of HSF5 (Figure 8). During pachynema progression, HSF5 interacts with SMARCA5, SMARCA4, SMARCE1 to repress the expression of certain genes (such as *sycp1*, *Meiob*, *Msh4* and *Hat1*), causing their transcription to decrease during their functional processes. Additionally, during mid- to late pachytene, HSF5 directly or indirectly promotes the expression of genes including *Hspa2*, *Ccnb1* and *Plk1*, preparing for the next step of desynapsis. Through these dynamic regulations of gene expression, HSF5 facilitates pachynema progression and ensures proper exit from pachytene.

## Discussion

Meiosis is a highly intricate process characterized by numerous concurrent or sequential steps requiring coordination by a plethora of regulators. During meiotic prophase I, the pachytene stage is recognized as the lengthiest phase, persisting for approximately six days in mice (27). Extensive investigations have elucidated pivotal features of pachynema, including complete synapsis alongside crossover formation, XY body formation, and MSCI (69–71). Nevertheless, our understanding of the protein regulatory network or key factors governing the entirety of pachynema progression remains limited. In the present study, we have identified HSF5 as a meiotic chromatin-associated protein, previously acknowledged as a lesser-known regulator within the HSFs family. HSF5 owned specific expressional pattern in male germ cells through pachynema progression. The absence of HSF5 resulted in a gradual halt in progression from mid- to late pachynema, ultimately leading to spermatocyte apoptosis and male infertility. Therefore, HSF5 plays an indispensable role in the completion of pachynema progression. Through the KO of *Hsf5*, our investigation has contributed a significant component to the intricate and enigmatic physiological process of pachynema progression.

In *Hsf5*^−/−^ mice, normal repair of DSBs, formation of the XY body, establishment of MSCI, and crossover formation were observed. However, the progression of pachynema was arrested, distinguishing it from previously reported mutant mouse models wherein pachynema arrest stemmed from defects in specific above events. For instance, deletion of *Mlh1* led to crossover deficiency, while *γH2AX*^−/−^ and *Mdc1*^−/−^ mutant males exhibited failure in XY body formation (72–74). Ultimately, spermatocytes in *Hsf5*^−/−^ mice progressed to cytologically mid- or mid-to-late pachytene stages but were unable to proceed to pachytene exit or desynapsis. These findings collectively suggest that HSF5 may not be directly involved in the homologous recombination process, but its deficiency impedes and halts pachynema progression, ultimately leading to cell death. While this paper was under review, Yoshimura et al. published similar findings. They detected partial γH2AX and MLH1 foci abnormalities in *Hsf5* KO pachytene cells, contrary to our data showing relatively normal foci in these cells (24). Additionally, both results exhibited normal XY body formation and MSCI, partially disagreeing with previous findings that showed elongated abnormal XY bodies and failure of MSCI with upregulation of sex chromosomal genes (23). We hypothesize that the discrepancies among these studies' findings could result from variations in sample preparation, staining, and photography. Despite these minor differences, above three studies conclude that meiotic arrest occurs at pachytene stages upon deficiency of *Hsf5*, and illustrate the underlying mechanisms from different perspectives, collectively elucidating the role of HSF5 in pachytene progression. Especially, our study provides a more detailed analysis at the single-cell level and examines gene transcription dynamics, offering additional valuable data and insights.

During meiosis, different transcriptional programs are activated sequentially and turned off timely to prevent conflicts between the previous and ongoing biological processes, and these regulators function in a step-by-step manner during the process, thus ensuring meiosis progression. Several studies have reported that transcriptional programs in meiotic prophase I are turned off through multiple mechanisms at different levels (12), such as epigenetic silencing of gene expression, mRNA degradation (75), or the degradation of proteins by the ubiquitin-proteasome degradation system (76). Our multi-omics integrative analysis reveals that during pachynema, HSF5 acts as a transcriptional suppressor of specific genes essential for pachynema progression, achieved through interactions with SMARCA5, SMARCA4 and SMARCE1 and binding to specific genomic regions. Although *Hsf5*^−/−^ mice exhibited seemingly normal mid-to-late pachynema through cytological analysis, their transcriptome significantly deviated from that of corresponding stages in *Hsf5*^+/+^ mice. Of particular significance is the considerable disruption observed in the transcriptional dynamics of key genes driving pachynema progression, resulting in the persistence of high transcript levels for proteins expected to gradually diminish during pachynema, such as *Sycp1*, *Msh4*, *Tesmin*, *Meiob*, *Hat1*. Overall, above results suggest that the chromatin-associated protein HSF5 may function as a transcriptional suppressor of specific pachynema progression driver genes through its interaction with the ISWI and SWI/SNF complex.

Further investigation is also required for the following aspects. Firstly, HSF5 exhibited high enrichment at the XY body during prophase I and was concentrated in a peri-chromocenter area in round spermatids (Figure 1E, J, K). However, we did not observe any obvious abnormalities in XY chromatin-associated biological events. Due to pachytene arrest in *Hsf5*^−/−^ mice, we were unable to explore its function in spermatids or spermiogenesis. Secondly, although HSF5 appeared to be an atypical member of the HSFs (17,54), our CUT&Tag results revealed that HSF5 efficiently bound to specific genomic regions of HSPs and HSF2 genes by recognizing the HSE or nnGAAnn motif. Additionally, *Hspa2*, bound by HSF5 (Figure 5D), is reported to be also required for spermatogenesis (11), exhibiting a phenotype extremely similar to that of *Hsf5*^−/−^ mice, wherein cytologically normal pachytene cells displayed impaired desynapsis. Such phenomena hinted special roles of HSF5 in the HSFs family and HSP-related biological regulation. Thirdly, HSFs have been previously recognized to trigger the expression of genes encoding heat shock proteins that act as molecular chaperone to the establishment of a cytoprotective state under various proteotoxic stresses and in several pathological conditions, indicating a conserved role of HSFs in inducing gene expression (18). In our present study, mild alterations in the transcriptional dynamics of HSF5-bounded induction driver genes were observed in *Hsf5*^−/−^ pachynema, irrespective of whether they encode HSPs, maintaining low transcription levels without an increase over the inferred cell time. These findings suggested that HSF5 may play a role in inducing certain gene expression in meiosis to some extent. Although we did not uncover concrete evidence to validate whether HSF5 promotes the transcription of these genes, its potential role should not be overlooked in future investigations.

## Supplementary Material

gkae701_Supplemental_Files

## Data Availability

The raw scRNA-seq datasets of mouse whole testis (fastq format) have been deposited to NCBI-SRA with the accession number PRJNA1098350. The raw CUT&Tag datasets of mouse whole testis (fastq format) have been deposited to NCBI-SRA with the accession number PRJNA1097357. The source code required to reproduce the single-cell analysis results of the article is provided, and intermediate files in the analysis are also available on Zenodo at https://doi.org/10.5281/zenodo.12530476. The raw protein spectrum data uploaded to the ProteomeXchange database can be accessed by the code PXD053613.
