# Supplementary material for: Meiotic chromatin-associated HSF5 is indispensable for pachynema progression and male fertility
Source: Nucleic Acids Res. 2024 Aug 20;52(17):10255–75. doi: 10.1093/nar/gkae701 (PMC11417359; doi:10.1093/nar/gkae701)
Supplement: gkae701_Supplemental_Files [file gkae701_supplemental_files.zip › Supplementary Materials .pdf]

Supplementary Figure

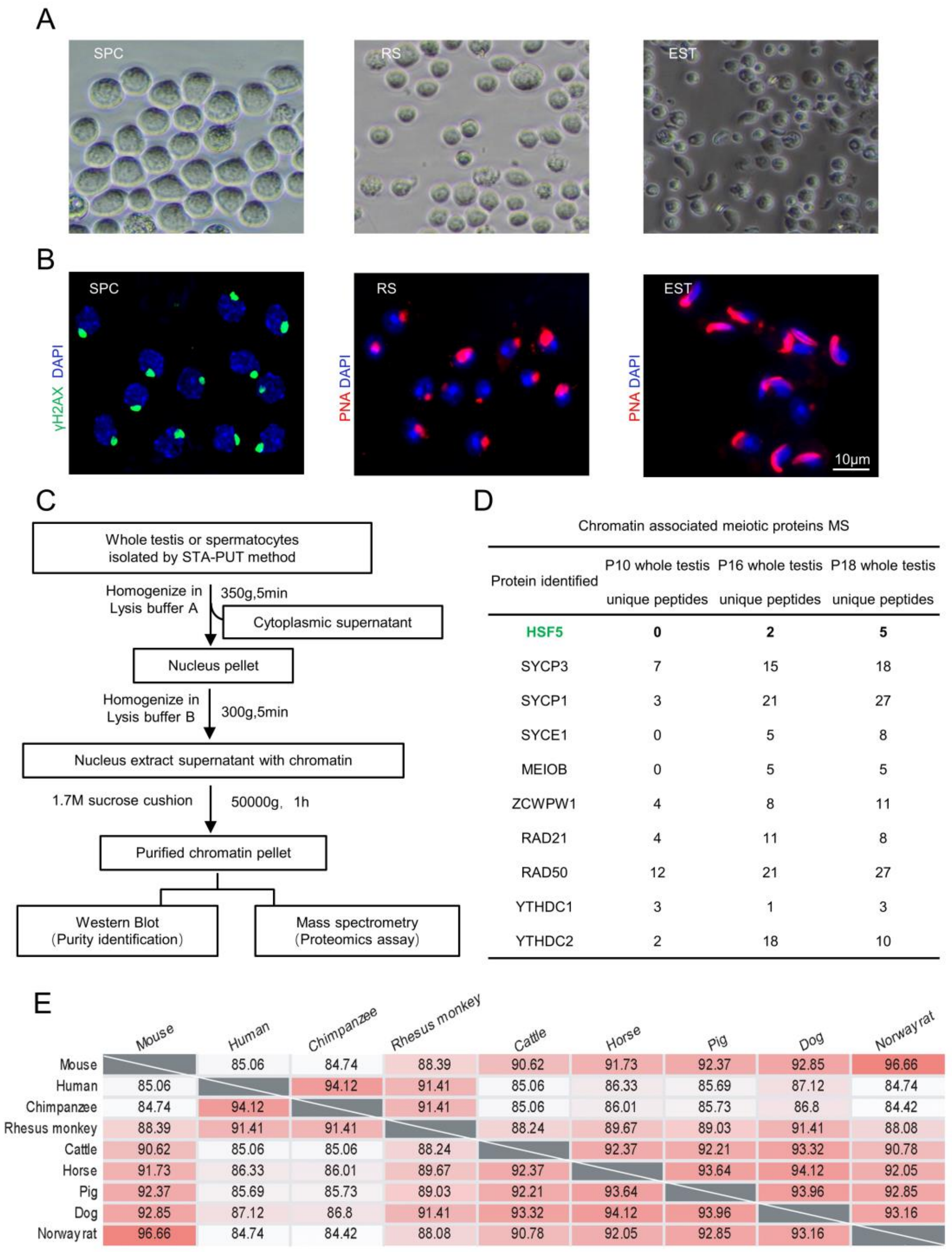

Supplementary Figure S1. Identification of a chromatin-associated and highly conserved protein-HSF5. (A) Morphology analyses of purified pachytene spermatocytes (SPC), round spermatids(RS), and elongating spermatids(EST). Scale bar is indicated. (B) Immunofluorescence staining of specific marker protein γH2AX

and PNA for isolated pachytene spermatocytes (SPC), round spermatids (RS) and elongating spermatids (EST). DNA was counterstained with DAPI. Scale bar is indicated. (C) Biochemical purification of chromatin. (D) Selective chromatin-associated protein candidates identified by MS analysis from developing testes at postnatal day 10(P10), P16, and P18. (E) A high degree of conservation of HSF5 in amino acid sequences among 9 species.

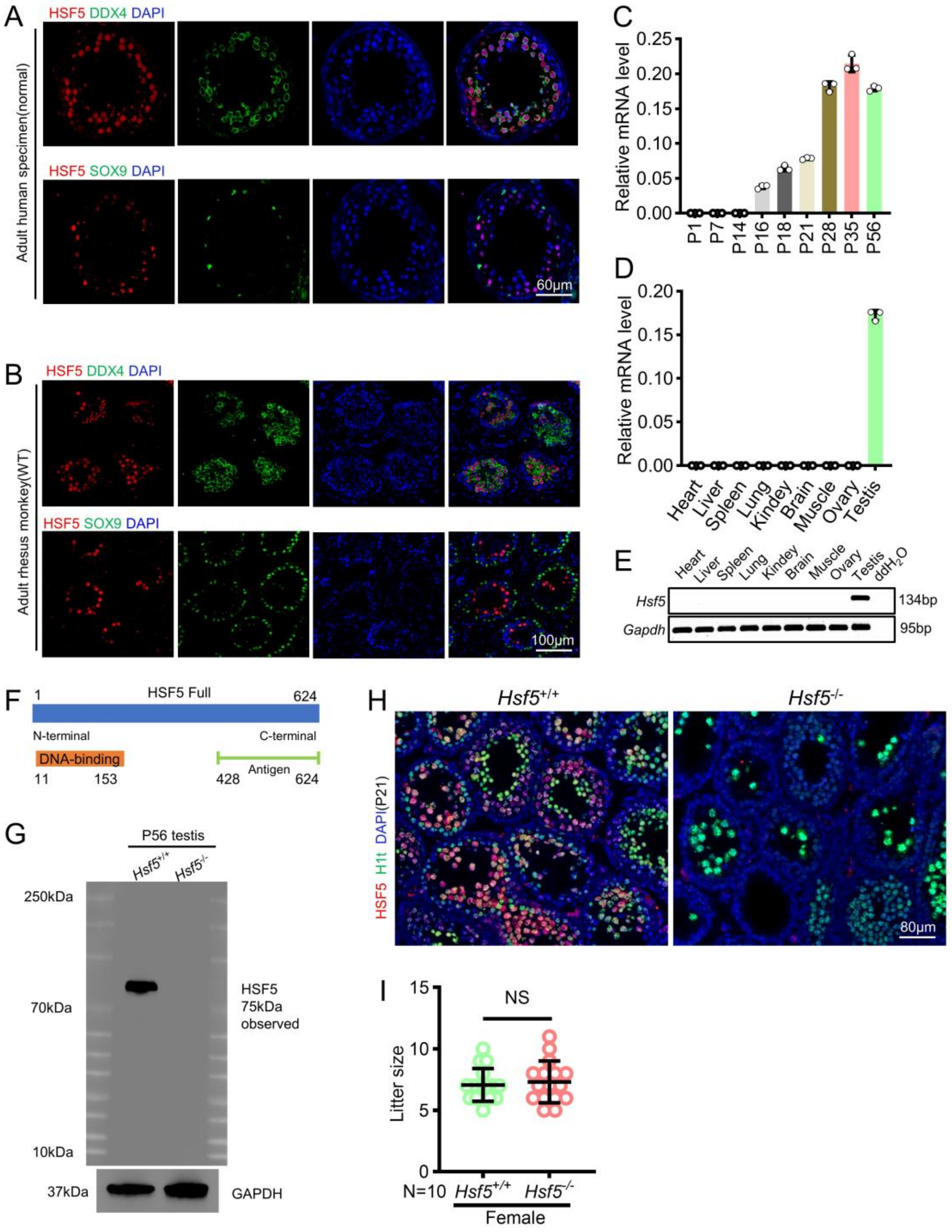

Supplementary Figure S2. Expression of HSF5 during testicular development and spermatogenesis in different species; HSF5 polyclonal antibody generation and validation. (A and B) DDX4 and SOX9 staining with HSF5 on testes sections at adult human(A) and Rhesus monkey(B) . Scale bars are indicated. (C and D) Analyses of *Hsf5* mRNA levels by qRT-PCR in developing testes at P1, P7, P14, P16, P18, P21,

P28, P35, P56 and in multiple organs or tissues in mice. *Gapdh* mRNA expression levels were used as the loading control. Data are mean±s.d. and were obtained from three independent experiments. (E) RT-PCR analysis of *Hsf5* gene expression in different organs or tissues. *Gapdh*, loading control. (F) Diagrams to illustrate full length, DNA binding domains(DBD), and polypeptide selected for the preparation of polyclonal antibodies of HSF5. (G) WB indicated a 75kDa protein in P56 *Hsf5*$^{+/+}$ mouse testicular lysates using homemade polyclonal antibodies. GAPDH, loading control. (H) H1t staining with HSF5 on *Hsf5*$^{+/+}$ and *Hsf5*$^{-/-}$ testes sections at P21. Scale bar is indicated. (I) The litter size of adult *Hsf5*$^{+/+}$ females and *Hsf5*$^{-/-}$ females. N, number of females. Data are mean±s.d. NS, non-significant (Unpaired Student's t-test).

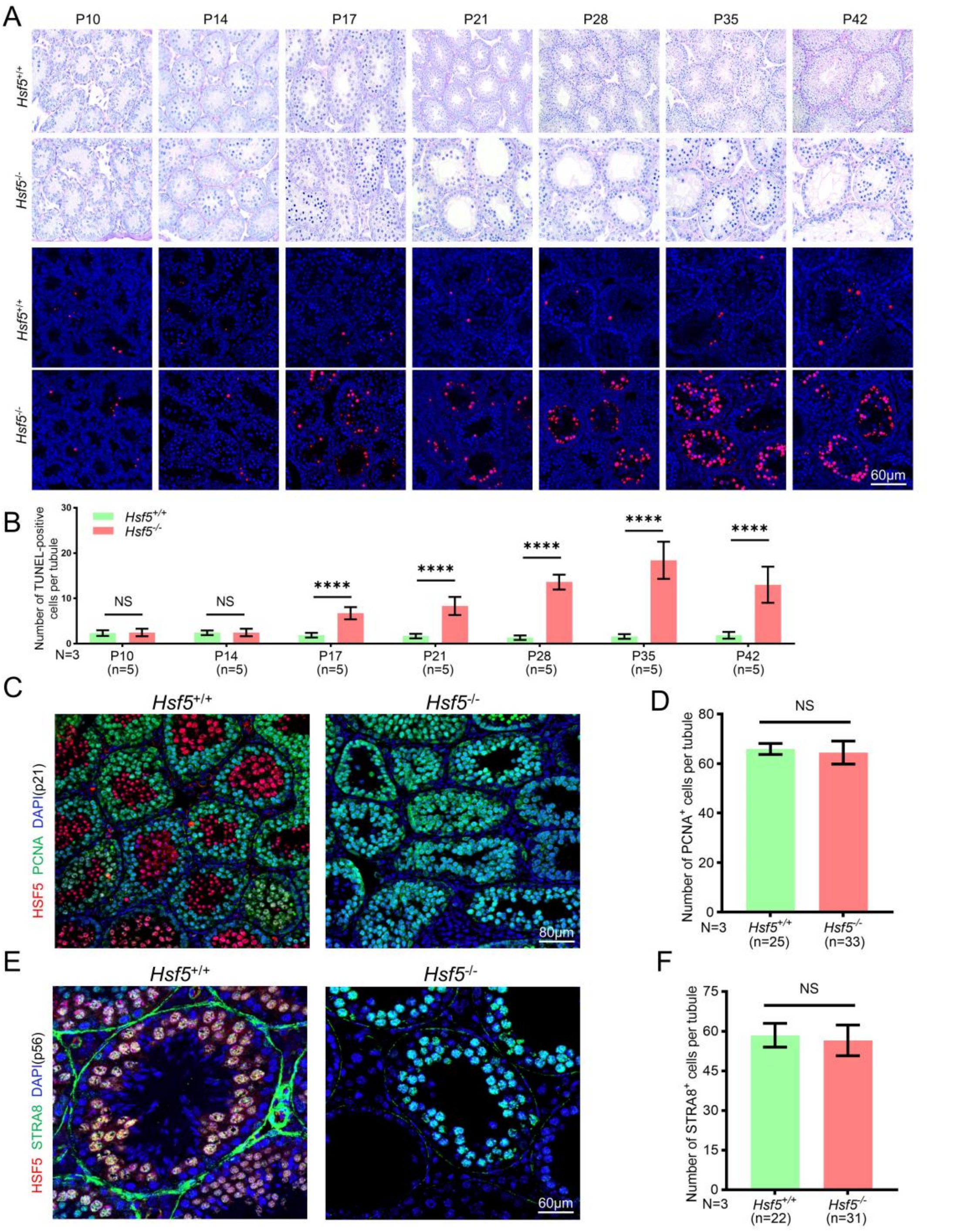

Supplementary Figure S3. Histological, TUNEL, differentiation, and meiotic initiation analyses on developing testes of *Hsf5*$^{+/+}$ and *Hsf5*$^{-/-}$ mice. (A) Representative PAS staining and TUNEL of developing testes at P10, P14, P17, P21, P28, P35 and P42 from *Hsf5*$^{+/+}$ and *Hsf5*$^{-/-}$ mice. Note that the morphology and total number of germ cells are distinguishable between *Hsf5*$^{+/+}$ and *Hsf5*$^{-/-}$ testes started from P17.

Scale bar is indicated. (B) Quantification of apoptotic cells in *Hsf5*$^{+/+}$ and *Hsf5*$^{-/-}$ developing testes at P10, P14, P17, P21, P28, P35 and P42. Scale bar is indicated. N, number of males; n, number of TUNEL-positive tubules analyzed. Data are mean±s.d. ****P<0.000 (Two–way ANOVA). (C and D) Quantitative comparison of PCNA immunofluorescence staining of testicular sections in P21 *Hsf5*$^{+/+}$ and *Hsf5*$^{-/-}$ mice. Scale bar is indicated. N, number of males; n, number of tubule cross sections analyzed. Data are mean±s.d. NS, non-significant (Mann–Whitney U-test). (E and F) Quantitative comparison of STRA8 immunofluorescence staining on testicular sections in P56 *Hsf5*$^{+/+}$ and *Hsf5*$^{-/-}$ mice. Scale bar is indicated. N, number of males; n, number of tubule cross sections analyzed. Data are mean±s.d. NS, non-significant (Mann–Whitney U-test).

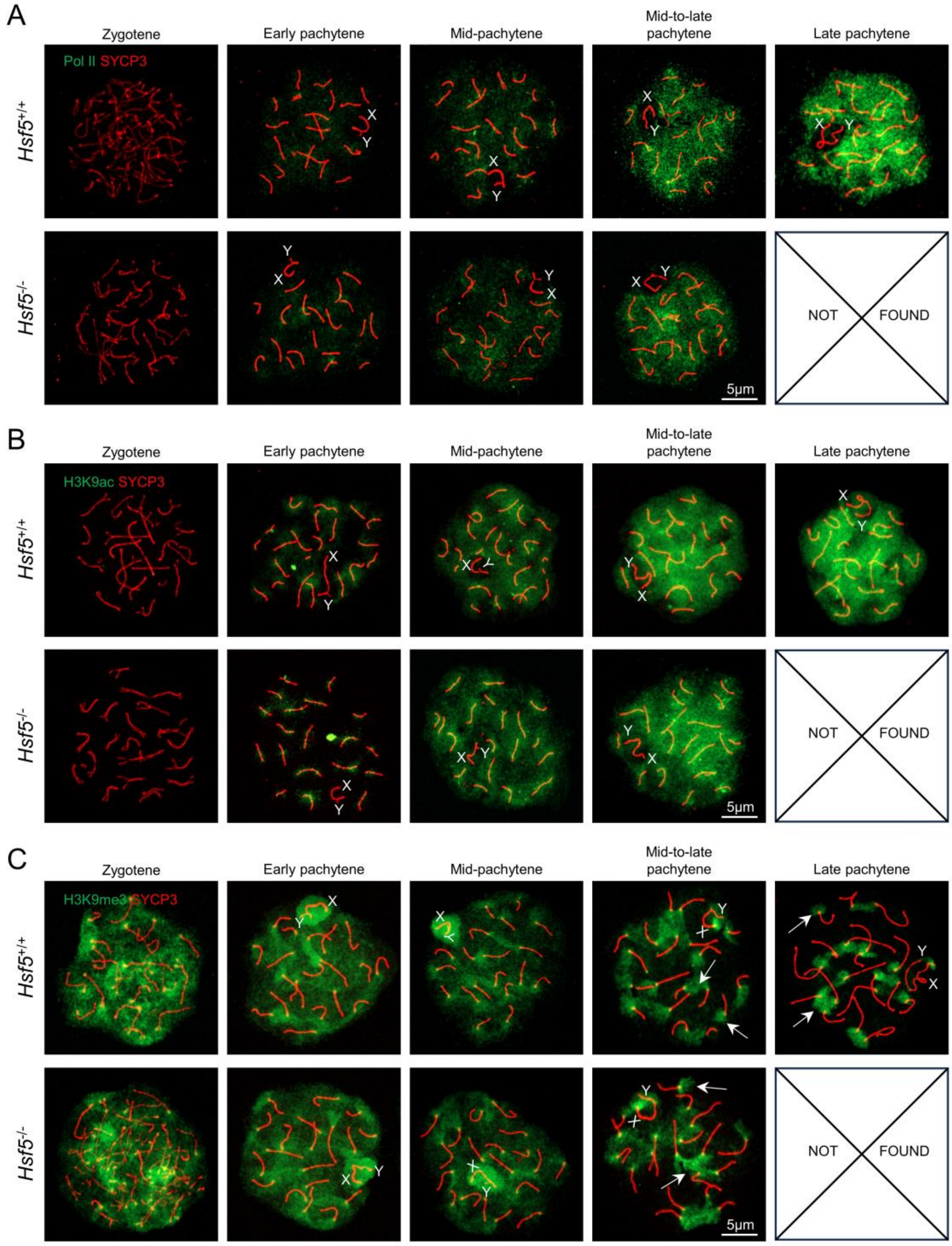

Supplementary Figure S4. *Hsf5*$^{-/-}$ pachynema exhibits unaffected MSCI identified by Transcriptional activity relative marker. (A-C) Zygotene to Late pachytene spermatocytes from P56 *Hsf5*$^{+/+}$ and *Hsf5*$^{-/-}$ immunostained for SYCP3, Pol II, H3K9ac, and H3K9me3. No discernible difference were found during prophase I between *Hsf5*$^{+/+}$ and *Hsf5*$^{-/-}$. Scale bars are indicated.

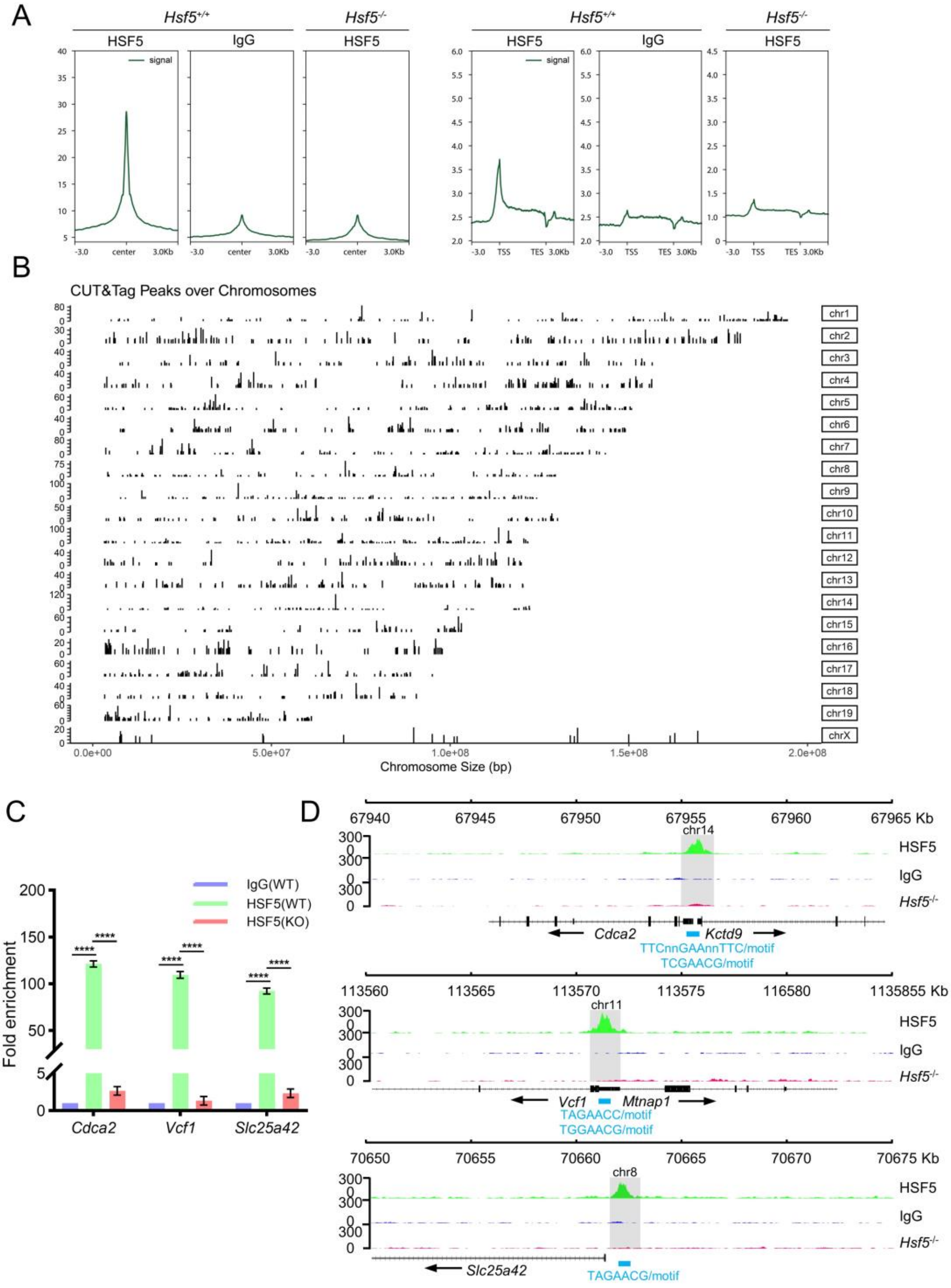

Supplementary Figure S5. Characteristics analysis of the HSF5-binding site. (A) Average distribution of HSF5 CUT&Tag signals around peaks, with all peaks aligned based on their highest signal point (left panel), and average distribution of HSF5 CUT&Tag signals around gene bodies (right panel). (B) Chromosomal distribution of HSF5 CUT&Tag peaks. (C) CUT&Tag-qPCR verified the enrichments of HSF5 at the

promoters of *Cdca2*, *Vcf1*, and *Slc25a42* in *Hsf5*$^{+/+}$ and *Hsf5*$^{-/-}$ pachytene spermatocyte. *Gapdh* promoter was used as a negative control. Data are mean±s.d. and were obtained from three independent experiments. ****P<0.0001 (Two–way ANOVA). (D) The track plot of CUT&Tag signals around the TSS of genes with high signal levels to HSF5, with the positions of HSE motifs marked at their genomic locations.

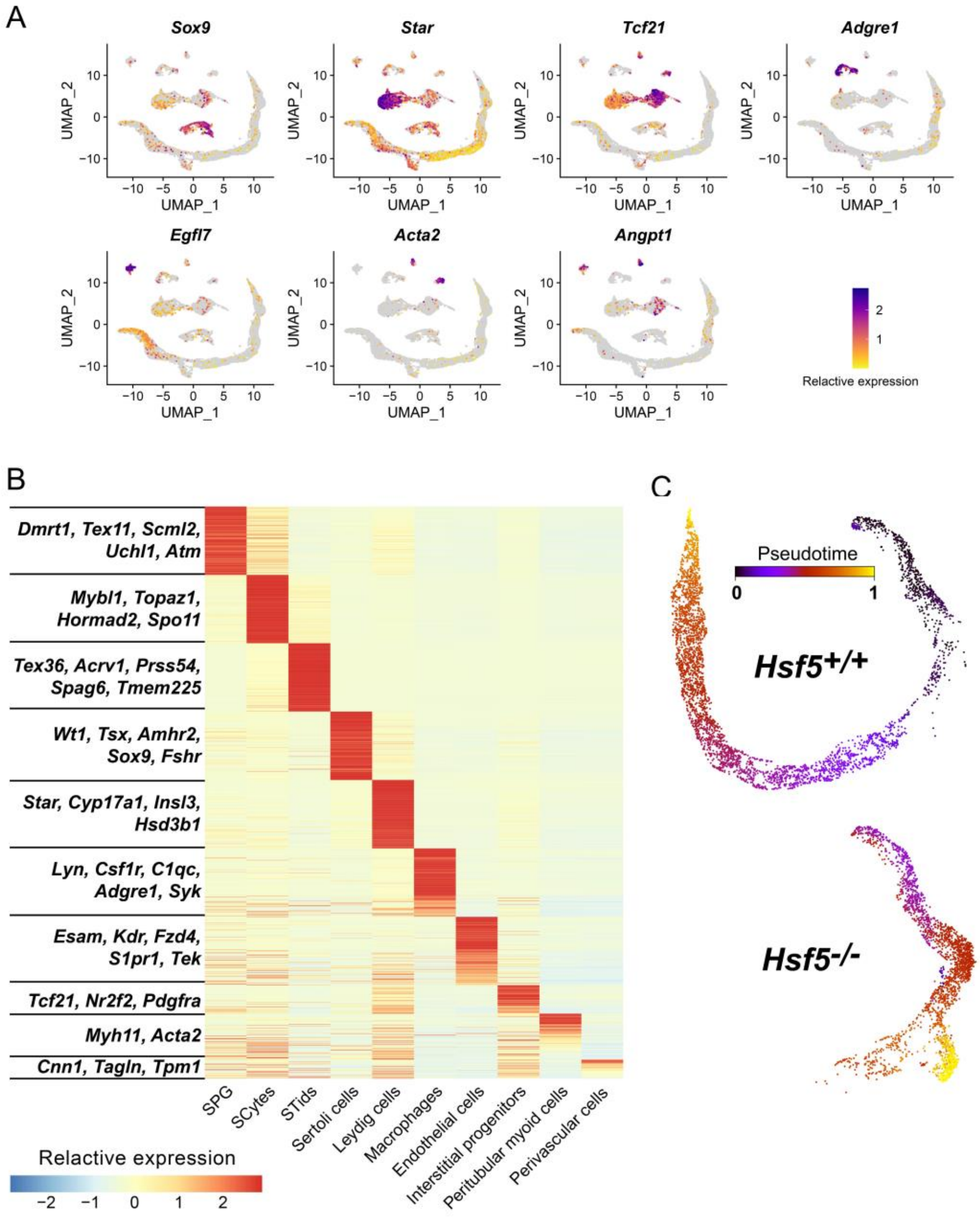

Supplementary Figure S6. Other characteristic analyses of scRNA-Seq in *Hsf5*$^{+/+}$ and *Hsf5*$^{-/-}$ related to Figure 6. (A) The representative gene expression patterns in somatic cells are demonstrated, with each gene's expression scale being independent of each other. (B) The heatmap displays the expression of representative genes across somatic cell clusters and preliminarily identified germ cell clusters. Some representative genes for each cluster are marked. (C) Velocity pseudotime analysis of germ cells from two samples. Each cell is colored according to its velocity time.

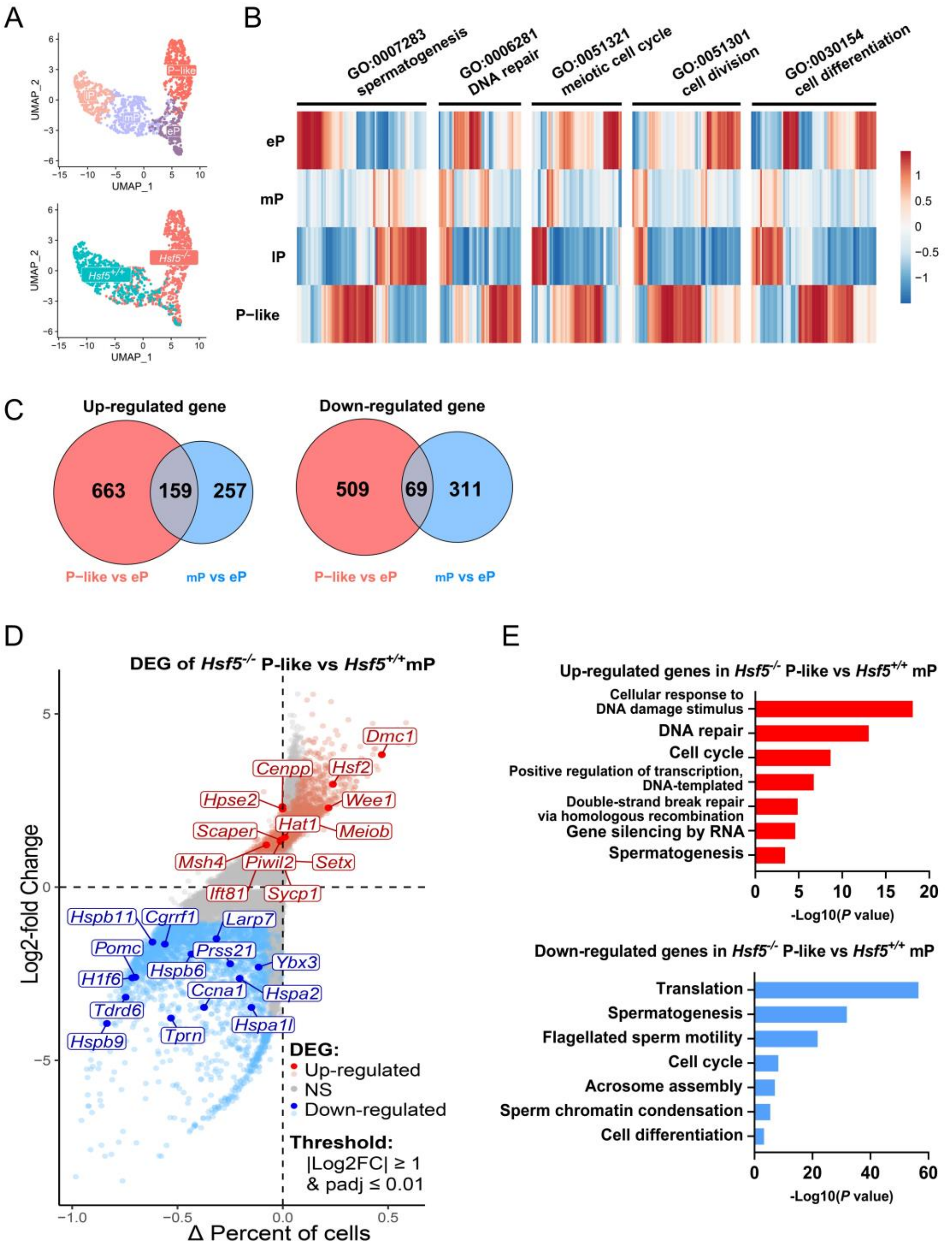

Supplementary Figure S7. Other characteristic analyses of scRNA-Seq in *Hsf5*$^{+/+}$ and *Hsf5*$^{-/-}$ related to Figure 7. (A) Re-UMAP analysis on the single-cell transcriptomes of pachytene stage cells from *Hsf5*$^{+/+}$ (n = 569 cells) and *Hsf5*$^{-/-}$ (n = 798 cells) mice. (B) Representative Gene Ontology (GO) terms of the biological process categories enriched in DEGs between eP, mP, lP, and P-like cluster cells. (C)

The number of up- and down-regulated genes in P-like/eP of *Hsf5*$^{-/-}$ mice or mP/eP of *Hsf5*$^{+/+}$ mice. (D) Differential gene expression analysis using the log2-fold change expression versus the difference in the percentage of cells expressing the gene comparing *Hsf5*$^{-/-}$ P-like cells versus *Hsf5*$^{+/+}$ mP cells (Δ Percentage Difference). The threshold for differential genes is log2-fold change > 1 and adjusted P-value from model-based analysis of single-cell transcriptomics (MAST) < 0.01. (E) Representative Gene Ontology (GO) terms of the biological process categories enriched in DEGs between P-like cells in *Hsf5*$^{-/-}$ mice and mP cells in *Hsf5*$^{+/+}$ mice.

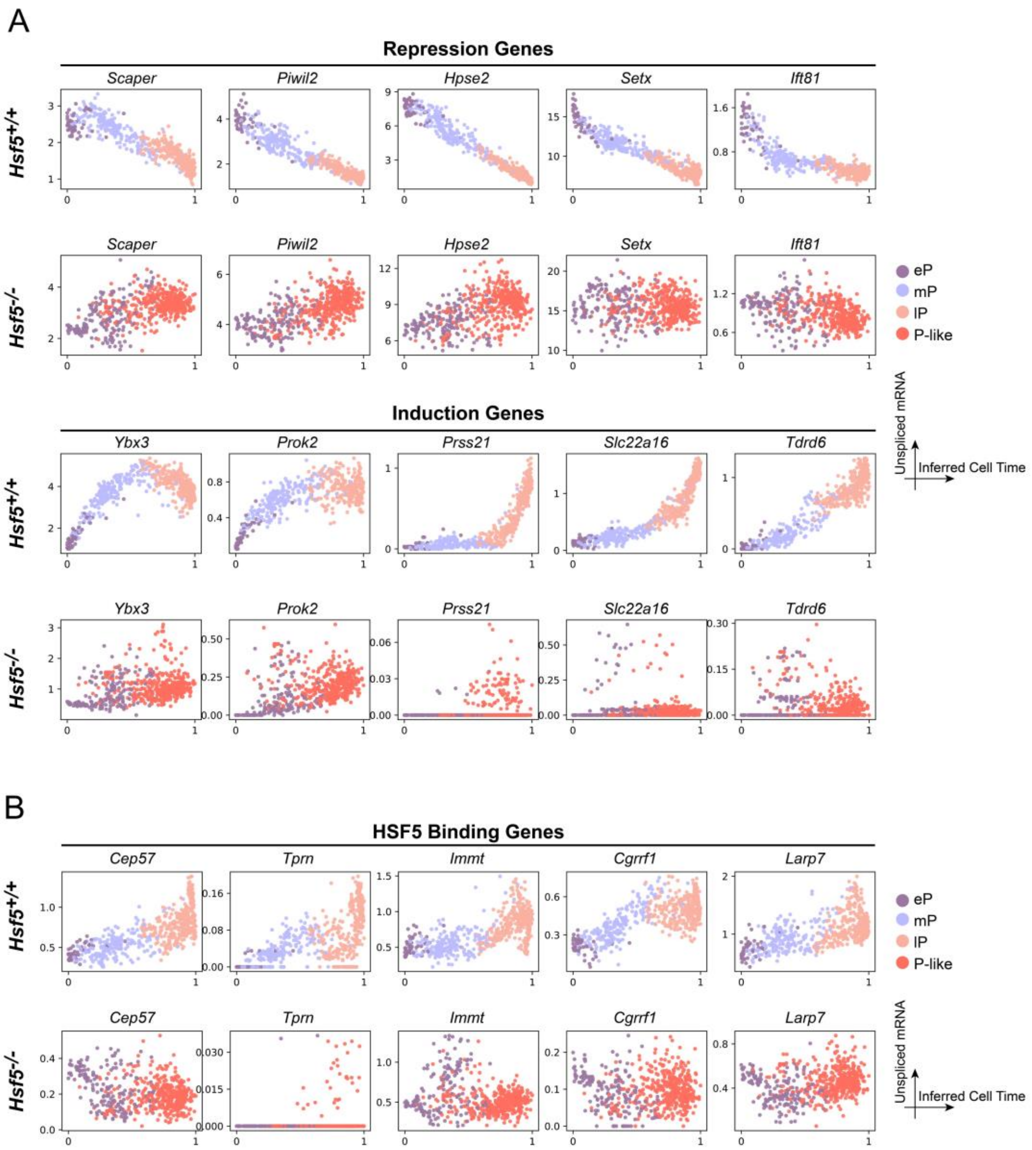

Supplementary Figure S8. Other characteristic analyses of scRNA-Seq in *Hsf5*$^{+/+}$ and *Hsf5*$^{-/-}$ related to Figure 7. (A) The changes in unspliced mRNA levels of driver gene at single-cell resolution along the inferred cell time. Each dot represents a single cell and is colored according to its cluster identity. The values along the x-axis and y-axis represent the order of cells along the inferred cell time and the unspliced mRNA levels of the gene, respectively. (B) The changes in unspliced mRNA levels of driver gene binding by HSF5 at single-cell resolution along the inferred cell time.
